# Recent advances in the biosensing platforms for sepsis diagnosis

**DOI:** 10.1039/d5ra06963g

**Published:** 2026-05-18

**Authors:** Vishwesh Dutt Mishra, Mohit Pandey, Hemant Kumar, Pradipta Kumar Panigrahi, Shantanu Bhattacharya

**Affiliations:** a Microsystem Fabrication Lab, Indian Institute of Technology Kanpur Kanpur U.P. India bhattacs@iitk.ac.in; b Department of Mechanical Engineering, Indian Institute of Technology Kanpur Kanpur U.P. India; c Department of Design, Indian Institute of Technology Kanpur Kanpur U.P. India

## Abstract

Sepsis is one of the major causes of mortality and organ losses due to the infections caused before or after hospitalization. According to the World Health Organization (WHO), 11 million people died from sepsis in 2020, which accounted for 20% of all deaths reported worldwide in the same year. This was out of an estimated 48.9 million cases of sepsis that year. Thus, the timely detection and treatment of sepsis are crucial for saving lives and organs of patients. There has been a continuous effort to identify and quantify the microbial load as well as ensure detection at an early stage of infection for ensuring the right treatment. There are various methods for identifying blood-based pathogens, and each of them has their own sensitivity and limit of detection (LOD). Identification by electrochemical and optical methods is unique in terms of their need and condition to be used. In remote and resource-deprived locations, paper-based, microfluidic, electrochemical or easy-to-use devices based on less-energy-intensive methods are more useful. In this work, we present the recent developments and challenges in the field of sepsis-related biosensors. The developments in neonatal-related sepsis biomarkers and sensors, the pathophysiology of sepsis, and the six categories of sepsis biosensors, with the relevant biomarkers, limits of detection (LOD), ease of extraction from bodily/interstitial fluids or analytes and their sensing mechanisms are discussed. The sensing mechanism plays a crucial role in deciding the reliability and durability of such sensors. The challenges in finding biomarkers directly from blood and preserving them while maintaining the integrity and viability for detection are discussed here. With the advent of the new era of artificial intelligence and machine learning, various diagnostic processes and techniques based on these technologies are developed, which are also discussed. This study presents a comprehensive analysis of different sensing techniques, with a particular focus on their sensitivity and limit of detection (LOD), and new mechanisms to simplify the process of pathogen detection with minimal processing time and process innovation.

## Abstract 2

Sepsis is one of the major causes of mortality and organ losses due to the infections caused before or after hospitalization.

## Graphical abstract

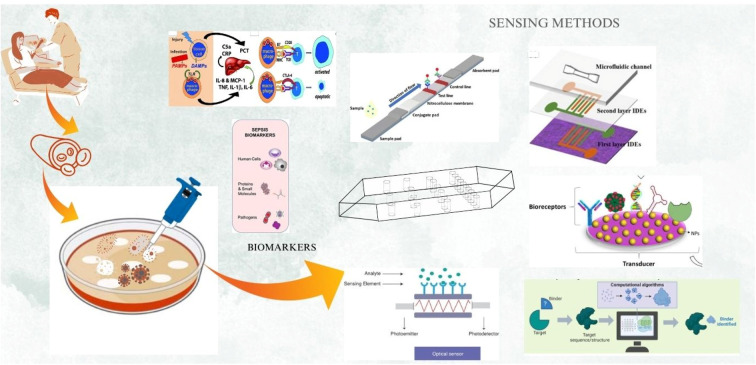

## 1 Introduction

**Fig. 1:**
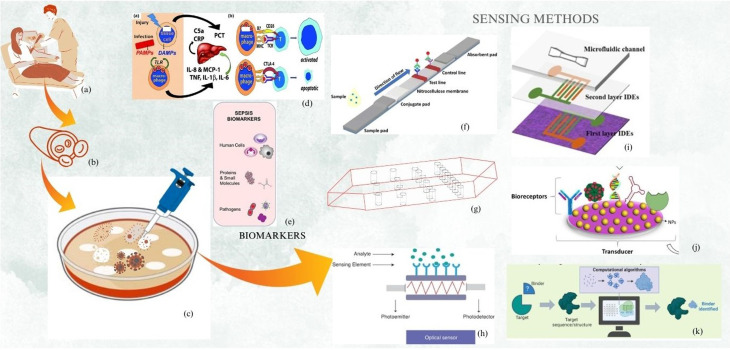
The schematic representation of sensing methods used for sepsis detection, showing (a) the blood extraction from patient, (b) the distribution of RBC and pathogen in vasculature, (c) the isolation of the biomarkers out of biofluids, (d) the action of sepsis-induced inflammation activity leading to affecting organs, (e) the crucial biomarkers associated with sepsis infection, (f) schematic of lateral flow assay based detection method, (g) schematic of microchannel flow, (h) schematic of optical sensor, (i) schematic of impedance based sensing, (j) schematic of Nanoparticle based capturing, (k) schematic of bioreceptor based & aptameric biosensing. Adapted from J. D. Faix (*Crit. Rev. Clin. Lab. Sci.*, 2013).^3^ B. K. Ashley, U. Hassan (*Wiley Interdiscip. Rev.:Nanomed. Nanobiotechnol.*, 2021).^4^ G. Bhatt *et al.* (*Sens. Actuators, B*, 2019).^5^ U. Jain, N. Chauhan, K. Saxena (Multifaceted Bio-sensing Technology, 2023);^6^ N. Idil, B. Mattiasson (IOP Publishing, 2021);^7^ M. B. C. Rashiku *et al.* (*IEEE SENSORS*, 2023);^8^ C. D. Flynn, D. Chang (Biosensors, 2023).^9^ Copyright © respective publishers.

The word sepsis originates from the Greek word ‘σηψις’, which refers to the bacterial decomposition of animal- or plant-based organic materials.^1^ The unregulated control of the defense mechanism of the body against an infection leads to a problem which may prove to be fatal, known as sepsis, and is turning out to be more dangerous than lung, bowel, and breast cancer, as in the last year it caused the deaths of 48 000 people. In the USA, every year, around 1.7 million people develop sepsis, resulting in the death of nearly 0.27 million people. The low-income countries are burdened with 85 sepsis patients out of every 100 individuals.^2^ India is also facing sepsis on a large scale; as in 2017, there were a total of 11 million patients and 3 million deaths due to sepsis.^2^ Therefore, the early-stage detection of sepsis that requires only a short analysis time becomes crucial. Developing or underdeveloped countries suffering from resource constraints along with the scarcity of trained human resources require simple methods of detection, which do not require much human intervention. The detection of sepsis using various bodily/interstitial fluids or analytes requires sensors to interact with them, resulting in an output that indicates the condition of patient (Fig. 1).

The problem of sepsis can occur in any of the two steps when an individual is not well. The rise in cases of anti-microbial-resistant bacteria is a major cause of organ failure and death; only in the USA, more than 50 000 individuals die due to such bacterial infections.^10^ Sepsis occurring during hospitalization is also a major cause of death, *i.e.*, around 17% in the USA. Therefore, immediate detection and early treatment with suitable antibiotics become important, otherwise cascading effects can lead to severe problems. Current gold standards like blood culture, urine culture, and sputum culture may take up to 5 days for results. After culture, the samples are tested for antibiotic susceptibility to identify the species. The gold standard for diagnosing sepsis is the culture of biofluids. Due to antibiotic administration, 40% of culture shows negative results. PCR (polymerase chain reaction) detects target DNA but fails to find antibiotic susceptibility. False-positive results may be obtained due to the confusion between host and contaminant DNAs.

Globally, 31.5 million people develop sepsis each year. Of these, 19.4 million experience severe sepsis and 5.3 million dies.^11^ Estimates suggest an incidence of 3 million cases of sepsis worldwide per year in neonates and 1.2 million cases per year in children, with mortality rates of 11–19%.^11^ Furthermore, more than 75 000 women die each year due to puerperal sepsis around the world. In hospitals in the United States, sepsis is not only the most expensive condition to treat but also the leading cause of death, with some reports estimating as many as 3.1 million cases at a cost of US $24 billion per year and mortality rates between 20% and 50%.^12,13^ Frustratingly, little progress has been made in the past three decades of the development of diagnostics and therapeutics for sepsis. Perhaps the main reason for this lack of progress is the vast heterogeneity in the immune response of sepsis patients, which has made the development of effective immunotherapies and the prediction of which infection cases will lead to life-threatening organ dysfunction difficult. Neonatal sepsis possesses a unique challenge due to low sampling volume for detection, and they cannot be pierced with needles for biofluids, and blood culture is risky to use due to prior antibiotics taken by the mother. Therefore, ICU patients, antibiotic resistance and neonatal cases present unique challenges for sepsis detection.

The prediction of mortality risk and its use in sepsis clinical trials have been done by Wong *et al.* In this approach, the group developed a Pediatric Sepsis Biomarker Risk Model (PERSEVERE), which performed well in diverse cohorts with septic shock. PERSEVERE biomarkers found that mice having a high risk of mortality have greater chance of bacterial infection, thus requires spikes in antibiotics. The problem of sepsis affects all age groups and all types of gender, hence timely intervention and treatment are necessary.^14^

To answer these questions, three tests are performed in series: a test for the presence of bacteria (typically by bacterial culture and growth), a test for pathogen identification (sometimes preceded by Gram staining) and antibiotic susceptibility testing (AST). From the onset of infection to the pathogen identification, it takes around 10 days in laboratory setup. Therefore, the need for POC (point-of-care) devices has become crucial. PCR helps in identifying the pathogen. There are two main diagnostic needs in sepsis management: pathogen information and host-response information. Host-response information can be gathered by measuring a variety of biomarkers, including but not limited to RNA, miRNA, plasma proteins, cell counts, cell-surface proteins, and small molecules, and mechanical properties, motility properties and other properties of cells. These biomarkers are involved in the progression of sepsis pathophysiology.

The major mechanisms driving sepsis progression combines the collective failure of cardiovascular, coagulation, cellular and endothelial dysfunction and causes multiple organ failure.^15^ Another major cause of ROS generation is oxidative stress, leading to endothelial dysfunction. Another cause of ROS generation is mitochondrial dysfunction damaging endothelial cells and causing inflammation.^16^ Sepsis is largely caused by bacteria, followed by viruses and then fungi, respectively. The most frequent infection causing bacteria were *Escherichia coli* (7.35%), *Streptococcus* (4%), methicillin-resistant *Staphylococcus* (2.86%) and *Staphylococcus* (1.9%). In 1991 the most frequent infective bacteria were *Escherichia coli* (7.35%), *Streptococcus* (4%), methicillin-resistant *Staphylococcus* (2.86%) and *Staphylococcus* (1.9%).^15^ Again in 2001, in the International Sepsis Definitions Conference, many factors like inflammatory, hemodynamic, organ dysfunction and tissue perfusion parameters were included in the diagnosis of sepsis. Between January 2014–2015 the European Society of Intensive Care Medicine and the Society of Critical Care Medicine assembled the data of “Sepsis-3”. Sepsis-3 symbolises organ dysfunction caused by disorganized host responses to infection, associated with SOFA score of 2 or more and symptoms like respiratory rate more than or equal to 22, hypotension.

To study the prevalence of sepsis in the Indian cohort, Todi and group conducted a study from August 2022 to July 2023 on suspected or confirmed infection cases, and SOFA scores of 2 or more were obtained for 19 ICUs. In this study, they found that the mortality rate was higher, *i.e.*, 36.3%, but shock mortality was 50.8% comparable to the west. In the Indian context, a majority of patients have community-acquired infections with the most common infection in the lungs. Tropical infections (dengue, malaria, and typhus) have very small contributions (2.2%), while major contributions come from Gram-negative bacterial strains (*Klebsiella* spp. (25%), *Escherichia coli* (24%) and *Acinetobacter* spp. (11%)).^17^

Surviving Sepsis Campaign International Guidelines for Management of Septic Shock, 2016, presents a meeting in which the surviving Sepsis Guideline Panel provided 93 statements on early management and resuscitation of patients with sepsis or septic shock.^18^ The GRADE (Grading of Recommendations Assessment, Development, and Evaluation) method is based on six categories: (i) risk of bias, (ii) inconsistency, (iii) indirectness, (iv) imprecision, (v) publication bias and (vi) other criteria (Tables 1–3).^19^

**Table 1:** Sequential organ failure assessment (SOFA) score. Reproduced from ref. 20 with permission from Springer Nature, Copyright 1996, Springer-Verlag.^20^

Measurable bodily parameters	Sequential organ failure assessment (SOFA) score	Mark
Oxygenation	PaO_2_/FiO_2_ >400	0
	=301–400	1
	<300	2
	=101–200 with ventilation support	3
	<100 with ventilation support	4
Coagulation	Platelets >150 k mm^−3^	0
	101–150 k mm^−3^	1
	51–100 k mm^−3^	2
	21–50 k mm^−3^	3
	<20 k mm^−1^	4
Blood pressure	MAP > 70	0
	MAP < 70	1
	ON dopa < 5 µg kg^−1^ min^−1^ or any dobutamine	2
	On dopa > 5 µg kg^−1^ min^−1^, epi < 0.1 µg kg^−1^ min^−1^ or NE < 0.1 µg kg^−1^ min^−1^	3
	On dopa > 15 µg kg^−1^ min^−1^, epi > 0.1 µg kg^−1^ min^−1^, or NE > 0.1 µg kg^−1^ min^−1^	4
Liver function	Total bilirubin < 1.2 mg dL^−1^	0
	Total bilirubin < 1.2 mg/bilirubin 1.2–1.9 mg dL^−1^	1
	Total bilirubin 2.0–5.9 mg dL^−1^	2
	Total bilirubin 6–11.9 mg dL^−1^	3
	Total bilirubin > 12.0 mg dL^−1^	4
Renal function	Cr < 1.2 mg dL^−1^	0
	Cr 1.2–1.9 mg dL^−1^	1
	Cr < 1.2 mg dL^−1^	2
	Cr 1.2–1.9 mg dL^−1^	
	Cr 2–3.4 mg dL^−1^	
	Cr 3.5–4.9 mg dL^−1^ or urine output < 500 mL d^−1^	3
	Cr > 5 mg dL^−1^ or urine output < 200 mL d^−1^	4
Level of consciousness	GCS 15	0
	GCS 13–14	1
	CGS 10–12	2
	CGS 6–9	3
	CGS < 6	4

**Table 2:** Quick sequential organ failure assessment (qSOFA) score. Reproduced from ref. 20 with permission from Springer Nature, Copyright 2016, the American Medical Association.^20^

Physical parameters	Parameter values (qSOFA = 1)
Respiratory status	Respiratory rate >22
Hemodynamics	SBP <100 mmHg
Mentation	Altered mentation (any degree)

**Table 3:** Body conditions for the early and simpler ways of infection assessment. Reproduced from ref. 21 with permission from Springer Nature; originally published by the American Medical Association, Copyright 2016.^21^

Temperature	>38 or <36 °C
WBC count	>12 000/mm^−3^ or <4000/mm^−3^
Heart rate	>90 bpm
Respiratory rate	>20 breaths/min
	PaCO_2_ <32 mm Hg

**Fig. 2:**
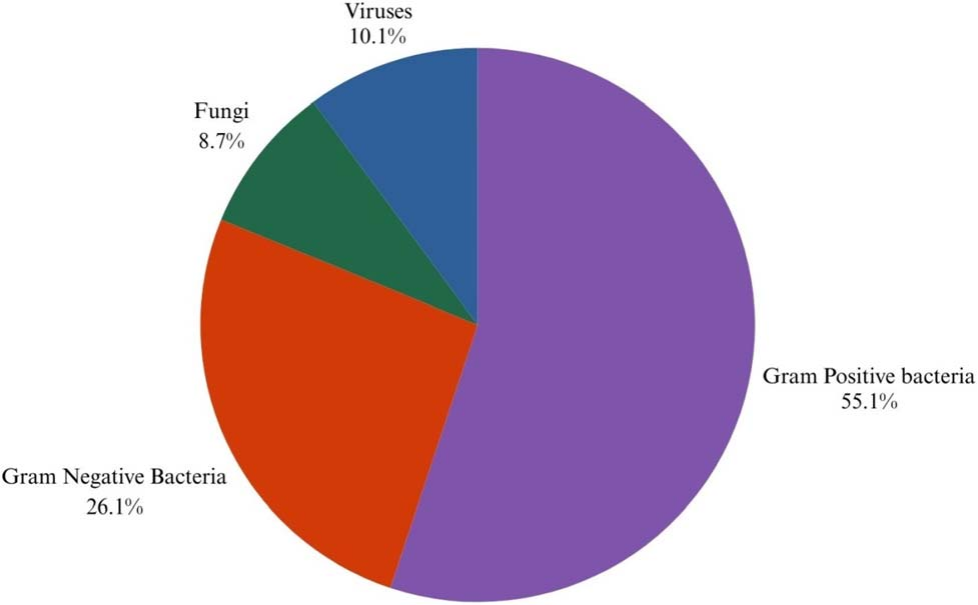
Pie chart representing the distribution of various pathogens responsible for sepsis.^26,27^

Sepsis-causing organisms can be broadly classified as viruses, fungi, and bacteria (either Gram-positive, Gram-negative, or mixed).^22,23^ Viral sepsis was extremely uncommon until 2020, when bacterial sepsis was the dominant entity. Since severe cases of COVID-19 can be classified as viral sepsis,^24^ the COVID-19 pandemic has drastically altered this. Although COVID-19-induced sepsis in intensive care units has significantly decreased since the pandemic, it is too soon to predict how this trend will play out in the future. The pathogens of bacteria, virus and fungi category which are responsible for the sepsis are given in Tables 4, 5 & Fig. 2.

**Table 4:** Table of pathogens responsible for sepsis

Bacteria	Gram-positive: methicillin-resistant *Staphylococcus aureus (MRSA)*, *Methicillin-sensitive Staphylococcus aureus (MSSA)*, *Staphylococcus epidermidis*, *Staphylococcus* spp., *Streptococcus pneumoniae*, *Streptococcus pyogenes*, *Streptococcus agalactiae*, *Streptococcus dysgalactiae* subsp. *equisimilis (SDSE)*, *Streptococcus anginosus*, *Streptococcus constellatus*, *Streptococcus* spp., *Enterococcus faecalis*, *Enterococcus faecium*, *Enterococcus* spp., *Clostridium difficile*, *Clostridium perfringens*, *Clostridium tetani*, *Clostridium* spp.
	Gram-negative: *Escherichia coli*, *Moraxella catarrhalis*, *Neisseria meningitidis*, *Neisseria* spp., *Acinetobacter baumannii*, *Acinetobacter* spp., *Aeromonas hydrophila*, *Aeromonas* spp., *Bacteroides fragilis*, *Bacteroides* spp., *Burkholderia cepacia*, *Burkholderia* spp., *Citrobacter freundii*, *Citrobacter* spp., *Enterobacter* spp., *Haemophilus influenzae*, *Klebsiella pneumoniae*, *Klebsiella oxytoca*, *Klebsiella* spp., *Legionella pneumophila*, *Legionella* spp., *Pseudomonas aeruginosa*, *Pseudomonas* spp., *Proteus mirabilis*, *Proteus* spp., *Rickettsia* spp., *Salmonella enteritidis*, *Salmonella* spp., *Serratia marcescens*, *Serratia* spp., *Stenotrophomonas maltophilia*, *Stenotrophomonas* spp., *Vibrio vulnificus*, *Vibrio cholerae*, *Vibrio* spp.^25^
Viruses	Herpes simplex virus, human parechovirus, enterovirus, influenza virus, dengue virus, adenovirus, SARS-CoV-2 (ref. 26)
Fungi	*Aspergillus* spp., *Candida albicans*, *Candida parapsilosis*, *Candida tropicalis*, *Candida krusei*, *Candida* spp.^27^

**Table 5:** Most common sepsis-causing bacteria

Bacterium	Sepsis type	Reference
*E coli*	Puerperal	28
*Klebsiella*	Neonatal	29
*Enterobacter*	Neonatal	30
*S. aureus*	Puerperal, septic shock & sepsis	31
*Streptococcus pyogenes*	Maternal sepsis	32

In this work, we discuss the biosensing methods, biomarkers, and biosensors developed by various techniques. These are various methods developed for sepsis diagnosis. First, we discuss the biomarkers for neonatal sepsis detection and the biosensing platform developed for them. After this, we present the key detection methods, *i.e.*, nanomaterial-based detection, lateral flow assay, electrochemical impedance spectroscopy, optical method, electrochemical method, microchannel-based approach which largely consists of microfluidics, newly developed machine learning-based diagnostics and aptamer-based detection. All the key methods are shown in Fig. 1.

Understanding the process by which the immune system leads to disease progression is important because it can help us in identifying the key biomarkers and knowing the level of severity of diseases. In sepsis infection various stages direct infection to septic shock and eventually death.

## 2 Pathophysiology of sepsis

**Fig. 3:**
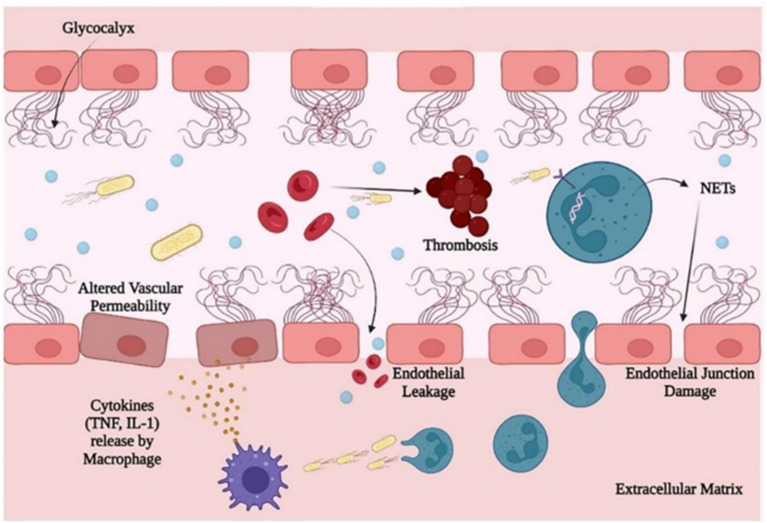
Schematic of the pathophysiology of sepsis, showing pathogen invasion triggering cytokine release (*e.g.*, TNF-α and IL-1), leading to endothelial activation, increased vascular permeability, and leakage. Neutrophil extracellular traps (NETs) and thrombosis further damage endothelial junctions, contributing to inflammation and organ dysfunction. (Reproduced with permission from Springer Nature. Copyright 2016, Springer Nature).^40^

The pathophysiology of sepsis begins with the combined study of primary hyperinflammation and immunosuppression, where hyperinflammation leads to sepsis and sepsis onsets begin with the ‘cytokine storm’.^33^ As shown in Fig. 3, pathogens attack the immune system, which in turn activates both the adaptive and innate responses. The pathogen attacks the host and initiates the response by recognizing the expression on receptors. TLRs (Toll-like receptors) detect PAMPs and DAMPs, and inflammatory response is generated.^33^ Moreover, NODs (Nod-like receptors) detect cytokine inflammation. Eventually, inflammation starts with the activation of leukocytes, coagulation and complement roadways that fortify the cellular, cardiovascular, and endothelial dysfunction.^34^ After this, the second stage of sepsis begins with the induction of apoptosis from an immune cell. Sepsis rapidly induces programmed cell like T-cells, NK cells, dendritic cells death showing nonspecific immunological responses.^34^ The complicated pathophysiology of sepsis obstructs the effective diagnosis and prophylaxis of the disorder. Soni *et al.* in their review presented the importance of endotoxin, Lipopolysaccharide (LPS) or endotoxin, a cell wall component of Gram-negative bacteria is crucial in pathogenesis of sepsis, its large amount om interacting with Toll-like receptor 4, generates abnormal rection by immune system.^34^ The root cause of sepsis infection lies not in microorganism inhibition but in the pathogen attack on body but due to dysregulated immune response while preventing the foreign organisms from spreading and multiplying inside the host body, resulting in multiple organ dysfunction, coagulopathy and hypotension.^35^ The inflated response of the immune system leads to microvascular thrombosis and organ dysfunction.^36^ Microvascular thrombosis can prevent pathogens from entering into the circulatory system from tissues, but this generalized phenomenon causes organ failure and eventually death due to extensive tissue ischemia.^37^ Boomer *et al.* conducted a study and found a massive apoptosis of T and B cells, which is accompanied by profound immunosuppression, which can turn out to be lethal.^38^ Adaptive responses act against specific pathogens or changes body cells, while innate responses are the first line of defense, which is fast but nonspecific. Innate responses provide protection *via* the skin, mucous membrane, immune system cells and proteins, while adaptive responses are specific and work by recognizing the infection type, and they help in fighting against germs if they attack again.^39^

Systemic inflammatory response syndrome (SIRS) is nonspecific early systemic inflammation; it is triggered by innate immune activation leading to high levels of proinflammatory cytokine, whereas specific immune dysregulation means dysregulated host response against infection leading to organ dysfunction. It involves both innate and adaptive immune system responses.^41^

In sepsis infection, immune cells undergo Warburg-like metabolic shift, inducing aerobic glycolysis even in the presence of oxygen. This produces inflammatory mediators, supporting early hyperinflammatory responses. A longer Warburg-like metabolic shift leads to immune exhaustion and tissue hypoxia.^41^

### 2.1 Stages of sepsis pathophysiology with biomarkers

Table 6 shows the stage of sepsis infection with respect to the biomarker, along with the pathophysiology and suitable biosensing platform, which has been developed. Sepsis is a complex interplay between the immune system, infection and multiorgan involvement. It becomes important to understand the role of each mechanism. Therefore, molecular signatures like cytokines and proteins can be key indicators in identifying the severity level and patient condition along with body immune conditions.

**Table 6:** Identification of the stages of sepsis using biomarkers

Stages	Pathophysiology	Representative biomarkers	Suitable biosensing platforms	References
Early recognition (0–6 h)	PAMP recognition, immune activation	LPS, presepsin, TNF-α, IL-6, miR-223	Aptameric, EIS, electrochemical, nanomaterial-based	42
Systemic inflammation (6–24 h)	Cytokine storm, endothelial activation	CRP, PCT, IL-8, MCP-1	LFA, electrochemical, optical	43
Dysregulation & coagulopathy (24–48 h)	Endothelial dysfunction, coagulation cascade	HMGB1, D-dimer, angiopoietin-2, miR-125b	Optical, microfluidic, EIS	44
Organ injury (>48 h)	Hypoxia, metabolic acidosis, organ failure	Lactate, NGAL, troponin-I, cfDNA	Electrochemical, microchannel, ML-integrated sensors	45

## 3 Traditional methods

### 3.1 ELISA

**Fig. 4:**
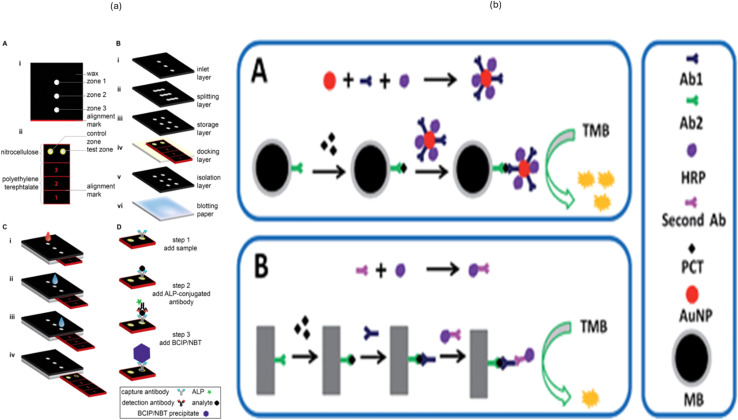
(a) Schematic of a multi-layered, paper-based microfluidic device, illustrating its design, fabrication, and operation. (A and B) Structural components, including a nitrocellulose sensing zone supported on a polyethylene terephthalate (PET) substrate, with hydrophobic wax barriers defining fluidic paths. The multilayer assembly comprises an inlet, splitting, reagent storage, docking, and isolation layers, stacked precisely using alignment marks. (C) Sequential sample processing steps: the addition of sample, binding of the analyte to immobilized capture antibodies, introduction of alkaline phosphatase (ALP)-conjugated detection antibodies, and subsequent addition of the BCIP/NBT substrate. (D) Formation of a visible blue precipitate as the colorimetric output, indicating the presence of the target analyte. This design enables low-cost, instrument-free, point-of-care diagnostics. (b) Signal amplification strategies in immunoassays using HRP-TMB chemistry, showing nanoparticle-assisted (A) and magnetic bead-based (B) detection mechanisms for enhanced sensitivity. (Reproduced with permission from Elsevier and the Royal Society of Chemistry, respectively. Copyright 2016, Elsevier Ltd; Copyright 2018, Royal Society of Chemistry).^48,49^

ELISA stands for enzyme-linked immunosorbent assay, which is used for antigen and antibody detection; since this method is very specific and costly at the same time, it is being replaced by other methods. Worthington *et al.* worked with forty patients with a clinical and microbiological diagnosis of intravascular catheter-related sepsis and positive blood cultures, caused by coagulase-negative staphylococci, and 40 control patients requiring a central venous catheter.^46^ Mahboob and group used membrane proteins, as they are potent antigens to detect anti-*P. multocida* antibodies, and *P. multocida* can be detected directly from blood through cell-based ELISA in dogs.^47^ Liao and group detected procalcitonin (PCT) based on magnetic beads and enzyme-antibody-labeled gold nanoparticles. This developed assay is sensitive enough to detect PCT at 20 pg mL^−1^, efficient for the direct human serum test.^48^ Notably, this assay specifically distinguishes PCT from other sepsis markers and the whole assay takes only 1.5 hours to finish. Another innovative design was developed by Verma *et al.*, who made a 3D µPAD to perform an enzyme-linked immunosorbent assay (ELISA).^49^ This device is highly sensitive and can detect CRP in the dynamic range of 1–100 ng mL^−1^ from a blood sample. However, it is limited by its accuracy of only 89% and a long assay time of 90 min. The key advantages of using 3D µPAD are simple fabrication, minimal instrumentation, low cost, and compact design. However, they have certain shortcomings like reagent use, short shelf-life, poor handling and lack of automation. The comparison of different methods with respect to blood culture & ELISA has been shown in Table 7 and Fig. 4.

**Table 7:** Difference in blood culture and other sensing methods

Parameter	Blood culture	ELISA	Other sensing method(s)
Detection principle	Growth of viable microorganisms	Antigen–antibody binding	Capturing or identifying different biological elements such as proteins, DNA, and RNA
Target element	Microorganisms	Specific biomarkers	Biomarkers, proteins, DNA, RNA, *etc.*
Biofluid	Blood		Any biofluid with biomarker or target microorganism
Load	Very low (gold standard)	More than blood culture	For most cases, higher than blood culture
Sensitivity	Highly sensitive	More than blood culture	For most cases, lower than blood culture
Detection time	High (2–3 days)	More than other methods but less than blood culture	Lower, usually in hours
Volume	Higher		Lower than blood culture
Cost	Low per test but high labor and time costs	Moderate	Higher initial cost, but low per-test consumables and rapid turnaround
Ease of use & automation	Need highly trained professional	Lesser	Most sensors can be used by common people
Multiplexing	Not possible	Limited	Possible
Specificity	Very high, even identifies the organism	High but cross-reactivity can occur	Based on the recognition element

### 3.2 Blood culture

The blood culture is used to isolate a microorganism for identification, susceptibility testing and typing. With technological advancement, blood is taken from the vein, and the blood sample is mixed with a culture in lab. The results of tests can be found within 24 hours, but the cause of infection will take up to 48–72 hours. If you get a “positive” result on your blood culture test, it usually means there are bacteria or yeast in your blood. “Negative” means there is no sign of them. Considered the gold standard for diagnosing sepsis, blood cultures can identify pathogens in the blood. However, blood cultures can be negative in up to 70% of patients with severe sepsis. Besides, urine tests are performed to investigate sepsis. An ELISA test can detect pathogen molecules and discriminate between patients with microbial infections and those with sterile trauma. Fast and precise identification of microorganisms in the early diagnosis of sepsis is crucial for enhancing patient outcomes. Digital PCR (dPCR) is a highly sensitive approach for absolute quantification that can be utilized as a culture-independent molecular technique for diagnosing sepsis pathogens. We performed a retrospective investigation on 69 ICU patients suspected of sepsis.^50^ Our findings showed that a multiplex dPCR diagnostic kit outperformed blood culture in detecting the 15 most frequent bacteria that cause sepsis. Ninety-two bacterial strains were identified using dPCR at concentrations varying from 34 copies per mL to 105 800 copies per mL. The detection rate of dPCR was much greater than that of BC, with 27.53% (19/69) *versus* 73.91% (51/69). The sensitivity of dPCR was 63.2%. Our research indicated that dPCR outperforms blood culture in the early detection of sepsis-causing microorganisms.

### 3.3 Blood coagulation

Traditional laboratory findings of sepsis including thrombocytopenia, increased prothrombin time (PT) and fibrin degradation products (FDPs), and decreased fibrinogen are the factors confirming the sepsis diagnosis especially due to the dysregulated host response rather than the infection itself. In the initial stages, the defense mechanism tries to stop the infection from spreading, but in advanced/serious stages, mass inflammatory cytokine production and release into the circulation lead to excessive activation of the coagulation process. The activation of coagulation is further enhanced by the release of inflammatory cytokines and antigenic products, and in advanced stages, the three anticoagulant pathways (antithrombin, protein C, and tissue factor pathway inhibitor) holding against the coagulation activation are deranged. Due to sepsis infection, antithrombic decreases, protein C level is suppressed, and these factor combinedly with other coagulation factors can be important biomarker for sepsis.^51^

## 4 Biomarkers

Biosensors are a subcategory of chemical sensors that utilize a biomolecule, cell, organism, or biological mimic in order to quantify a target analyte. The most common biosensor configuration involves modifying a transducer with a biorecognition element, which captures the analyte with high selectivity and specificity. This triggers a change in physical property that is measured by the transducer. A biomarker is a measurable quantity or substance found in body or bodily fluids which changes its form and concentration leading to pathogenic problems and defining deviation in normal and pathogenic or problematic condition.^52^ The bacteria that can cause sepsis or eventually septic shock are *Staphylococcus aureus* (20.5% of cases), *Pseudomonas* species (19.9%), *Escherichia coli* (16.0%), *Klebsiella* species (12.7%), *Enterococcus* (10.9%) and *Staphylococcus epidermidis* (10.8%), and the *Candida* fungus (17%) can also result in the same. These bacteria mainly affect the lungs (36–42%), genitourinary tract (10–18%), abdomen (8–9%) and wounds or soft tissues (7–9%), and in about 20% of cases, we are not able to identify the source of bacteria.^53^ 54 patients assessed in clinical studies, 23 in clinical and experimental studies, and 3 in only experimental studies biomarkers were identified and were added to the previously identified 178 biomarkers.^53^ The rise in C-reactive protein concentrations, neutrophilia and release of immature myeloid cells are observed.

Presepsin alone or in combination with other biomarkers helps in better ways for diagnosis and prognosis of sepsis in patients.^54^ Similarly, a study conducted by Hung *et al.* found CD-64 and presepsin as the most reliable biomarkers among others.^55^ Adrenomedullin, angiopoietin and the mid-regional fragment of pro-adrenomedullin and non-coding mRNAs can be useful for prognosis.^56^ Nakajima and group found PCT as a responsible or decisive factor by testing the serum of three groups formed. In addition, the level of PCT was found to be significant. Interestingly, they found no difference between the WBC values and CRP (C-reactive protein).^57^

**Fig. 5:**
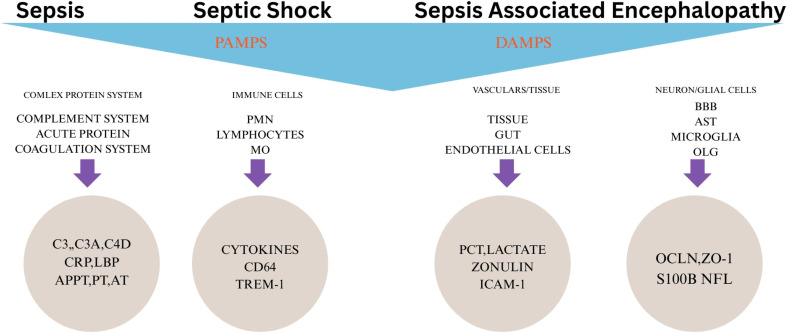
Overview of key molecular pathways and biomarkers involved in the progression from sepsis to septic shock and sepsis-associated encephalopathy (SAE). PAMPs trigger systemic immune and protein responses, while DAMPs contribute to tissue, endothelial, and neuronal damages, each associated with distinct biomarkers. Reproduced from ref. 59 under the terms of the Creative Commons Attribution-Noncommercial (CC BY-NC) License.

**Fig. 6:**
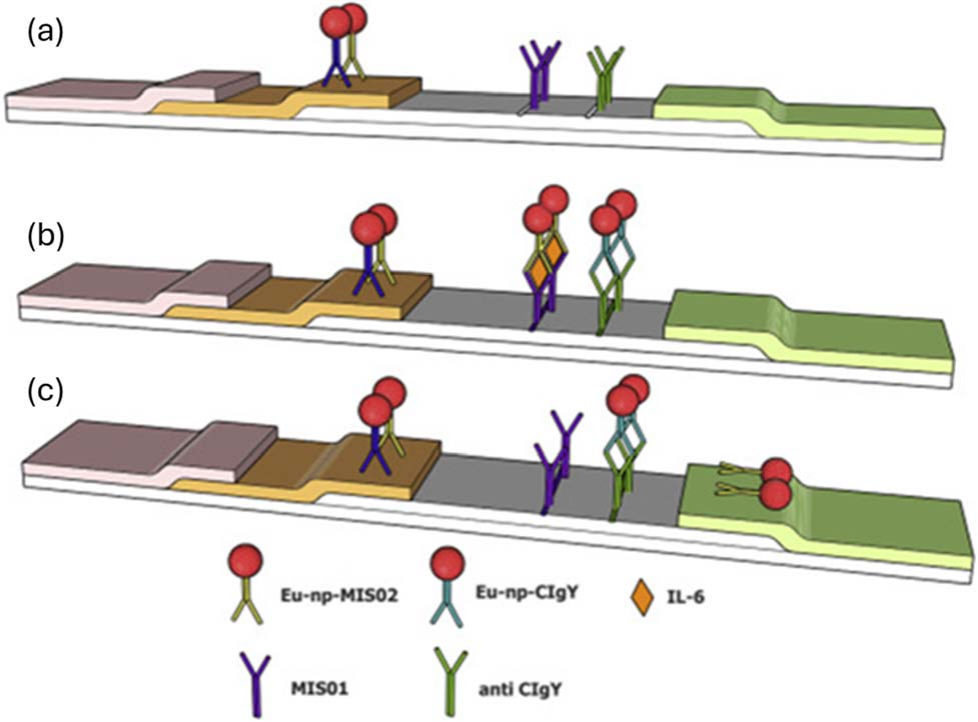
The schematic representation of paper-based assay, (from (a)–(c)), Multiplexed immunoassay on a microarray chip for sepsis biomarker detection. (a) Immobilization of conjugated antibodies (MIS01) on chip and europium nanoparticle-conjugated detection MIS02 antibodies (Eu-np-MIS02). (b) Sample containing IL-6 cytokines, which bind specifically to the immobilized MIS01 antibodies is introduced in channel. Simultaneously, Eu-np-CIgY antibodies bind to anti-CIgY capture antibodies. (c) Eu-np-MIS02 binds to IL-6 captured by MIS01, enabling fluorescence detection, and Eu-np-CIgY/anti-CIgY ensures reliability. (Reproduced with permission from Elsevier, Biosensors and Bioelectronics, 2016. Copyright 2016, Elsevier Ltd^105^).

MR-proADM is mainly used as a prognosis biomarker, since high levels of this molecule for prolonged periods of time have been associated with poor outcomes.^56^ Other biomarkers are CD14 (presepsin), sTREM-1 (soluble Triggering Receptor Expressed by Myeloid cells 1), copeptin, a peptide derived from preprovasopressin, and suPAR (soluble urokinase-type plasminogen activator receptor). Liver dysfunction (LD) occurs in 19% of cases of septic shock, so early-stage liver cell analysis becomes crucial in deciding the possibility of occurrence of LD. Therefore, Sauer developed a cell-based cytotoxicity biosensor, using the human cell line HepG2/C3A. Immunomodulators are medicines which increase or decrease the immune response to work suitably on cytokine storm.^58^ In Fig. 5, we show the organs affected by the associated biomarkers, and in Table 8, we show the biomarker and the associated LOD. In Table 9, we present a glimpse of some most commonly used biosensing methods and their corresponding target molecules. Fig. 6 shows the schematic of the lateral flow assay and microfluidic platform. However, these methods may become difficult to use in cases of multiple biomarker identification, since they usually focus on single analytes. Therefore, there is a need for multiplexing the diagnostic process to improve the overall efficiency.

**Table 8:** Biomarkers along with the limit of detection and samples used

Biomarker	Diagnostic targets	Sensing method	Source	Sample type	Role	Concentration	Peak type after stimulus	References
**Proteins**								
CRP, hsCRP	Inflammation intensity	Immunoassay, electrochemical	Liver	Blood, urine, sweat	Hyper inflammatory phenotype	<3 µg mL^−1^	4–6 hours	60–62

**Complement proteins**								
PTX-3	Inflammation intensity	Immunoassay, electrochemical	Endothelial cells, fibroblasts	Blood plasma	Differentiates sepsis & septic shock	5.24 ng mL^−1^	NA	63 and 64

**Cytokines & chemokines**								
IL-1β, IL-6, IL-10	Inflammation intensity	Immunoassay, electrochemical	Monocytes, endothelial cells, and adipose tissue	Cerebrospinal fluid & blood	Organ dysfunction prognosis & hypo inflammatory phenotype	<25 pg mL^−1^	6 hours	65–67
IL-8	Inflammation intensity	Immunoassay, electrochemical	Macrophages	Cerebrospinal fluid & blood	NA	<10 pg mL^−1^	1–3 h	68

**DAMPs**								
Calprotectin	Host tissue damage	EIS, SPR	Neutrophils	Serum, plasma, amniotic fluid, and ascitic fluid	Confirms sepsis infection & 30-day mortality	>4 mg mL^−1^	NA	66 and 69
HMGB-1	Host tissue damage	EIS, SPR	Chromosome 13	Blood	28-day mortality	NA	NA	44 and 70

**Endothelial cells and BBB markers**								
Ang-1, Ang-2	Sepsis-induced organ failure	Optical, impedance	Proteins	Blood, urine, CSF	Activating & blocking the Tie-2 receptor	(Ang-2/Ang-1) >1	NA	71
OCLN	Sepsis-induced organ failure	Optical, impedance	Epithelial cell lining	NA	Helps in finding SOFA score	>0.5 ng mL^−1^	NA	72 and 73
S100B	Sepsis-induced organ failure	Optical, impedance	Glial cell in brain	NA	Delirium in septic shock, sepsis-associated encephalopathy	0.01. µg mL^−1^	NA	74 and 75
E-selectin	Sepsis-induced organ failure	Optical, impedance	Surface of endothelial cell	NA	Predicts mortality, SOFA & APACHE-II	>16 ng mL^−1^	NA	76
sFlt-1	Sepsis-induced organ failure	Optical, impedance	Endothelial cells and placental cells	NA	Prognosis of sepsis severity, SOFA score	>190 pg mL^−1^	NA	76

**Gut permeability markers**								
Citrulline	Endotoxemia	Enzymatic, electrochemical	By product of urea process	Liver, kidney	Indicates early acute bowel dysfunction	<10.1 ± 2.9 µmol kg^−1^ h^−1^	NA	77 and 78
d-Lactic acid	Endotoxemia	Enzymatic, electrochemical	Gastrointestinal Tract	Liver	Early intestinal damage	>4 mmol L^−1^	NA	79
Non-coding RNAs					28-day mortality risk			
miR-125a, miR-125b	Early diagnosis and mechanistic insight	Nucleic acid hybridization, aptameric	Blood	Major organs and tissues	Risk of sepsis and increased mortality	NA	NA	80–82
Inc-MEG3	Early diagnosis and mechanistic insight	Nucleic acid hybridization, aptameric	NA	NA	Related to mortality	NA	NA	83

**Membrane receptors, cell proteins, and metabolites**								
CD64	Activation of neutrophils and monocytes	Aptameric, electrochemical	Monocytes	Blood	Early infection & 28-day mortality risk	<8 mg L^−1^	24 h	84
CD68	Activation of neutrophils and monocytes	Aptameric, electrochemical	Macrophages & Microglia	Blood	Microglial activation	NA	NA	85
NFL, NFH	Septic encephalopathy	Immunosensor	Plasma	Nervous system	Risk and severity of sepsis-associated encephalopathy	NA	NA	86
Presepsin	Activation of neutrophils and monocytes	Aptameric, electrochemical	Blood	WBC	Differentiates bacteria type	>582 pg mL^−1^	NA	87
TREM-1	Activation of neutrophils and monocytes	Aptameric, electrochemical	Neutrophils	NA	Early distinction between sepsis and SIRS	>60 ng mL^−1^	NA	88 and 89

**Peptide precursor of the hormone and hormone**								
PCT	Neuroendocrine stress	Microfluidic, immunosensor	Thyroid gland	Blood	Predicts bacterial infection & sepsis	<0.05 ng mL^−1^	12–24 hours	90
MR-proADM	Neuroendocrine stress	Microfluidic, immunosensor	NA	Blood	Organ dysfunction marker	1.8 nmol mL^−1^	NA	91

**Neutrophil, cells, and related biomarkers**								
Lactate	Endotoxemia	Enzymatic, electrochemical	Myocyte tissue	Blood, urine	Predicts mortality	<2 nmol L^−1^	24 h	92
MPO	Activation of neutrophils and monocytes	Enzymatic, electrochemical	NA	Neutrophils	Mortality predictor at 28- and 90-day septic shock	>60 ng mL^−1^	NA	93 and 94
Resistin	Inflammation intensity	Enzymatic, electrochemical	NA	Adipose tissue	Mortality predictor at 28-day & septic shock	NA	NA	95

**Soluble receptors**								
suPAR	Immune suppression/activation balance	Electrochemical, optical	NA	Blood	Predictive mortality at 7 and 30 days	NA	NA	90
sPD-L1	Immune suppression/activation balance	Electrochemical, optical	NA	Blood	28-day mortality predictor, immunosuppression	NA	NA	96

**Lipoproteins**								
LDL-C	Host lipid metabolism	Optical, nano-plasmonic	NA	Liver	Protective effect	NA	NA	97
HDL	Host lipid metabolism	Optical, nano-plasmonic	NA	Liver	Mortality prognosis, adverse clinical outcomes	NA	NA	98

**Table 9:** Common methods of detection and target molecules

Method of detection	Target molecule	Agent	Reference
Colorimetric detection	DNA, LPS, Cu^+2^	AuNPs, AgNPs	99 and 100
Immunoassay	CRP	AuNPs	101 and 102
Electrochemical reaction	IL-3	Magnetic NP	103
Magnetic separation	Lipopolysaccharide (LPS), endotoxin	Magnetic bead, Fe_3_O_4_-Ce6-Apt	104

## 5 Methods of detection

### 5.1 Neonatal sepsis and detection

**Fig. 7:**
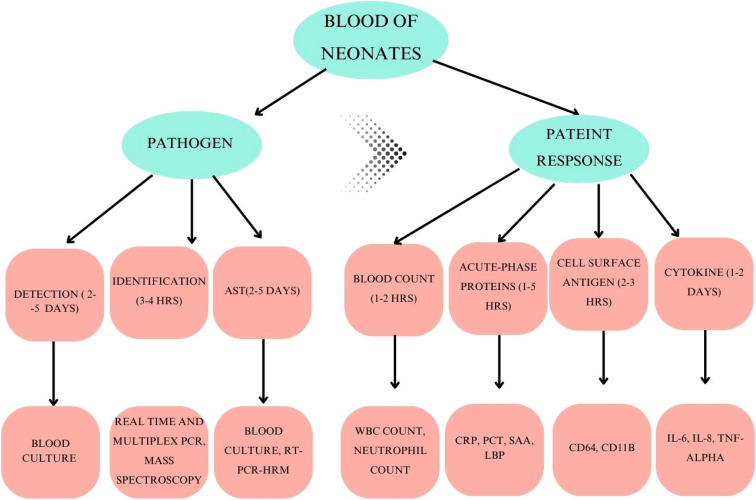
The schematic representation of neonatal sepsis infection detection using blood, associated cause and biomarkers, it also represents the pathogen and patients response along with presenting the associated process from detection to identification, and biomarkers associated with variables assessing the patient response. (Adopted with permission from Pediatric Research, 2017. Copyright © 2017 Springer Nature).

Neonatal sepsis, which occurs in infants within 28 days of birth,^106^ can be dangerous and even deadly. An important biomarker in such cases is such a definite pattern in problem and treatment along with requiring minimum amount of blood. Traditional biomarkers like cytokines and CRP are important, but the combination of biophysical and biochemical biomarkers makes the analysis more reliable. CD-4 level can be detected easily within first 6 hours itself, while Procalcitonin is at its peak 2–4 days but finding CRP may takes only 2 days to reach peak. Biophysical parameters like heart rate, respiratory rate, core temperature, body weight, number of desaturation events, and number of bradycardic events are important in detecting sepsis. Newborns gets infected by neonatal septicemia, caused by *Escherichia coli* and Group B *Streptococcus* (GBS). Gopal *et al.* devised an electrochemical sensor in which the electrode is coated with multi-walled carbon nanotubes (MWCNTs), manganese oxide nanospheres (MnO_2_NSs), and cobalt oxide nanoparticles (Co_3_O_4_NPs) to detect CRP, PCT and serum amyloid (SAA).^107^ The limit of detection was 0.01 pM to 1 µM. Gopal used a fimA gene of *E*. *coli* for neonatal sepsis detection and designed a 20-mer long amine-terminated oligonucleotide for bioreception. Their detection and sensitivity range were 10^−12^ M to 10^−6^ M and 114.7 µA M^−1^ cm^−2^.^108^ In Fig. 7 & Table 10, a holistic representation of neonatal sepsis and associated biomarkers is given.

**Table 10:** Neonatal sepsis biomarkers and limit of detection

Biomarker	Abnormal level	Detection limit & time	Reference
**C-reactive protein (CRP)**	>10 mg mL^−1^		
Printed electrode	NA	0.15 nM–17 ng mL^−1^ & 30 s	109
Immunoassay FETs	NA	0.1–100 ng mL^−1^ & 20 min	110
EC immunosensor	NA	2.2 ng mL^−1^ & 2 h	111
EC impedimetric	NA	176 pM & 15 min	111
Magnetic	NA	25 ng mL^−1^ to 2.5 mg mL^−1^ & 30 min	112
EC biosensor	NA	0.5–70 nM & 1 h	113
RNA aptamer-based	NA	100–500 pg mL^−1^	114
DNA aptamer-based	NA	1 pM & 30 min	115
Electrochemical	1–24 µg mL^−1^	NA	116
Giant magnetoimpedance	1–10 ng mL^−1^	NA	117
Optical	19.478 ng mL^−1^	NA	118
Electrochemical	37 nM, 0.1 µg mL^−1^	NA	119
Plasmonic	27 pg mL^−1^	NA	120
Optical imaging	0.1 pg mL^−1^	NA	121
Optical-fiber-optic	0.01 mg L^−1^	NA	122
Optical-refractive index	0.1–10 µg mL^−1^	NA	123
Optical-FL	27.8 pM	NA	124
Optical-SPR	2–5 µg mL^−1^, 50 ng mL^−1^	NA	125
**Procalcitonin (PCT)**	>1 ng mL^−1^	NA	
DNA aptamer	NA	0.5 ng mL^−1^ & 1 h	126
EC biosensor	NA	0.39 ± 0.11 nM	127
Electrochemical/non-faradaic	0.1 ng mL^−1^	NA	128
Electrochemical	0.09 ng mL^−1^, 0.39 ± 0.11 nM	NA	129
Optical-FL scanning	0.13 µg mL^−1^	NA	130
Optical-TIRF	0.04 ng mL^−1^	NA	130
Optical-SPR	3 pg mL^−1^	NA	131
Amperometric	0.8 pg mL^−1^	NA	132
Electrochemiluminescence	3.4 pg mL^−1^	NA	129
Optical-LSPR	11.29 pg mL^−1^	NA	133
**Serum amyloid A (SAA)**	187.6 ± 78.3 µg mL^−1^	NA	134
**Lipopolysaccharide-binding protein (LPBP)**	13.0–46.0 1 g mL^−1^	NA	135
**Tumor necrosis factor alpha (TNF-α)**	>6 ng mL^−1^	NA	42
EIS	NA	1–100 pg mL^−1^ & 30 min	136
**Interleukin-6 (IL-6)**	100 pg mL^−1^		
Potentiostatic capacitance	NA	5 × 10^−16^–5 × 10^−13^ M	137
Optical fibre ball	NA	0.91 fM, 273 aM to 59 fM	138
**Pentraxin 3**	43.06 ± 3.88 g L^−1^	NA	139
**Interleukin-8 (IL-8)**	>60 pg mL^−1^	NA	
EC impedance	NA	900 fg ml^−1^–900 ng mL^−1^ & 15 min	140
Au NW	NA	200 pM & 2 h	141
Optical-FL scanning	0.13 µg mL^−1^	NA	141
**Neopterin**	>32.2 nmol L^−1^	NA	
Printed electrodes	NA	0.44 ppb & 20 min	142
EC immunoassay	NA	0.008 ng mL^−1^ & 20 min	143
Optical-FL scanning	0.15 ng mL^−1^	NA	144
**TLR-4**			
EC endotoxin sensor	NA	0.0002 EU mL^−1^ & 30 min	145
**Fibronectin**			
Colorimetric	0.156 µg mL^−1^	146	
**Presepsin**	168.9–935.6 pg mL	1174–4854 pg mL	147

### 5.2 Nanomaterial-based biosensing

#### 5.2.1 Nanoparticle preparation

**Fig. 8:**
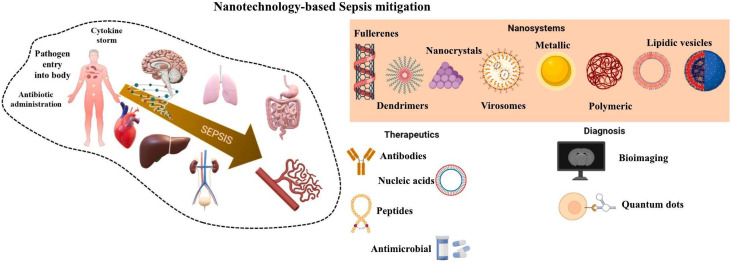
Schematic of the nanotechnology-based sepsis detection. Various nanotechnology-based approaches are shown for both therapeutic and diagnostic interventions. Nanocarriers such as fullerenes, dendrimers, nanocrystals, virosomes, metallic and polymeric nanoparticles, and lipidic vesicles are employed to deliver therapeutics including antibodies, nucleic acids, peptides, and antimicrobials. Diagnostic tools leverage bioimaging and quantum dots for the early detection and monitoring of sepsis. (Reproduced from Elsevier, *Journal of Controlled Release*, 2022. Copyright 2022, Elsevier B.V.).^40^

Nanoscale particles with metallic and polymeric compounds are now-a-days highly used for a wide variety of applications from environmental to advance battery materials.^148–152^ These nanoparticles along with biomarkers have now become a more frequently used technique in biosensors. The protein biomarkers, such as procalcitonin (PCT), C-reactive protein (CRP) and interleukin 6 (IL-6), will show variations in their levels as sepsis progresses, signifying their usage in its detection. Over the past few decades, a wide range of serum (or plasma) sepsis indicators have been commercialized as a result of these inherent constraints. These usually consist of neutrophil CD64 (nCD64), soluble triggering receptor expressed on myeloid cells-1 (sTREM-1), serum soluble urokinase-type plasminogen activator receptor (suPAR), procalcitonin, presepsin, interleukin 6 (IL-6), lipopolysaccharide-binding protein (LBP), and so on. Nevertheless, none of these biomarkers would carefully meet all the requirements to be considered a sepsis biomarker. A nanocomposite planer gold electrode was used to detect PCT, which gives high correlation with ELISA (*r*^2^ = 0.95), and a multiplex platform for PCT, C-reactive protein, and pathogen-associated molecular patterns is also developed.^153^ Janus particles (having distinct surface properties), which have not been used frequently were used by Russell *et al.*, here they have used Iron oxide as core to provide colour and magnetism and Janus as coating to convert motion into colors. This helped in avoiding nonspecific binding and capturing target molecules,^153^ which is shown in Fig. 8, Tables 11 and 12 present the combination of various nanomaterials with samples along with the methods of detection.

**Table 11:** Table of nanomaterials and the methods of detection

Technique	Nanomaterials	Sample	Working range	Limit of detection	Analysis time	Sample volume	Reference
DPV	MWCNTs	Serum	0.01–350 ng mL^−1^	0.5 pg mL^−1^	>1 h	NA	154
DPV	OMSi-Zn	Serum	0.05 pg mL^−1^–80 ng mL^−1^	0.013 pg mL^−1^	>1 h	NA	155
CV^c^	rGO	Blood (50%	0.09–10.24 ng mL^−1^	64.5 pg mL^−1^	7 min	50 µL	156
		Diluted blood)	0.07–2.49 ng mL^−1^	24.7 pg mL^−1^			
DPV	SWCNHs/HPtCs	Serum	1 pg mL^−1^–20 ng mL^−1^	0.43 pg mL^−1^	>40 min	NA	157
Amperometry	FeCN-AuNPs	Serum	1.5 pg mL^−1^–50 ng mL^−1^	0.8 pg mL^−1^	>1 h	100 µL	158
Amperometry	rGO-AuNPs	Serum	0.05–20 ng mL^−1^	0.1 pg mL^−1^	>1 h	v	159
SWV/amperometry	CuCo_2_S_4_–Au	Serum	0.0001–50 ng mL^−1^	82.6 fg mL^−1^		6 µL	160
				95.4 fg mL^−1^			
Amperometry	MBs	Plasma	0.25–100 ng mL^−1^	0.09 pg mL^−1^	<20 min	<30 µL	161
ECL	MOFs-FcaZOF8	Serum	5 pg mL^−1^–100 ng mL^−1^	0.85 pg mL^−1^	>1 h	6 µL	162
ECL	MOF-MIL-101	Serum	0.014 pg mL^−1^–40 ng mL^−1^	3.4 fg mL^−1^	24 h	10 mL	163
LSPR	AuNPs	Serum	4.2–12500 pg mL^−1^	2.8 pg mL^−1^	25 min	NA	164
AlphaLISA	Acceptor beads	Serum	0.016–100 ng mL^−1^	18.6 pg mL^−1^	0.5 h	5 µL	165
PPSc	NA	Serum	0.05–200 ng mL^−1^	10.64 pg mL^−1^	<1.5 h	NA	166
ABS	MNPs	Serum	1–10000 pg mL^−1^	0.045 pg mL^−1^	1 h	800 µL	167
Amperometry	MBs	Serum	0.07–1000 µg mL^−1^	0.021 ng mL^−1^	4.5 h	25 µL	
		Whole blood	0.005–1 µg mL^−1^	1.5 ng mL^−1^	15 min	5 µL	
LSPR	AuNHAs	Cell media with horse serum	NA	0.021 pg mL^−1^	2 h	NA	168
	AuNPs	NA	NA	100 ag mL^−1^	>1 h	NA	
	AuNHAs	Plasma	NA	18 µg mL^−1^	>1 h	NA	

**Table 12:** Microfluidic methods of sepsis detection

Microfluidic method	Targeted microorganisms/biomarkers	Sample	LOD	References
Acoustophoresis	*E. coli*	Peripheral blood mononuclear cells	10^6^ cell per mL	169
	*Pseudomonas putida*	Plasma	10^3^ cell per mL	170
	*P. aeruginosa*	Whole blood	120 cells per mL	171
Dielectrophoresis	*E. coli*	NA	10^6^ cell per mL	172
	*E. coli*	NA	10^4^ cells per mL	173
	*C. albicans* & *S. aureus*	NA		174
	*E. coli* and *S. aureus*	Whole blood	1000 cells per mL	174
Immunoaffinity	*E. coli*	NA	50 cells per mL	175
	*E. coli*	Flow with magnetic bead	10^5^ cells per mL	
	Gram-negative and gram-positive bacteria, *E*. *coli*	Blood	5 × 10^6^ cells per mL	176
	*C. albicans*	Whole blood	10^6^ cell per mL	177
Inertial focusing	*E. coli*	Blood	10^6^ cell per mL, 10^8^ cell per mL, 62% separation	178–180
	*E. coli*, *S. aureus*, *P. aeruginosa*, *Enterococcus faecalis*	WBC, RBC	10 cell per mL	181
Adhesion-based Methods	*E. coli*	Blood	10^7^ cells per mL	182
	*E. coli*	Blood	10^7^ to 10^9^ cells per mL	183
**Electrochemical**				
EC sensor with Au electrode	NA	Blood	176 pM	
EIS-based output	NA	CRP	11 ng mL^−1^	184
Amperometric	NA	Pctab2	0.03 pg mL^−1^	185
Multiplex	NA	Infected blood	290 CFU mL^−1^	186
Aptasensor	NA	TNF-α	0.58 nM	187
Immunosensor	NA	IL-6	0.01 pg mL^−1^	188
Immunosensor	NA	PCT	6 pg mL^−1^	189
**Optical**				
Photoelectrochemical	NA	IL-6	0.38 pg mL^−1^	190
Lateral flow	NA	CRP	27.8 pM	191
SPR	NA	PCT	4.2 ng mL^−1^	184
**Immunosensors**		NA		
Electro-chemiluminescence	NA	PCT	NA	192
Silica nanoparticle	NA	TNF-α	NA	193
Fluorescence	NA	Spiked blood	NA	194
**Field-effect transistor**	NA	CRP	0.1 ng mL^−1^	195
Organic FET	CRP	Saliva	590 zM	196
Colorimetry	PCT	Blood	0.4 ng mL^−1^	197
Electrolyte-gated OFET	PCT	Buffer	2.2 pM	198
FET	CRP	Buffer	0.1 ng mL^−1^	199
Quartz crystal microbalance—D300 QCM unit	Folate-binding proteins	Serum	50 pM to 2 µM	200
Amperometric magneto-immunosensor	NA	CRP	0.021 ng mL^−1^	201

Factors affecting the delivery and function of nanoparticles are as follows:

(1) Size – optimally sized NPs can avoid steric hindrance and sufficiently interact with cells.

(2) Shape – rod-shaped nanoparticles are of high priority for cell uptake.

(3) Charge – ensures stability in suspension

(4) Ligands – to ensure NP functionality.

Gold nanoparticles (AuNPs) are the most widely used nanoparticles for detection purposes due to their better conjugation and higher surface activities, leading to their widespread use in novel biomaterial synthesis and diverse applications. AuNPs were first synthesized by Gustav *et al.* using hydrogen tetrachloroaurate (HAuCl_4_) with citric acid in boiling water.^202^ This method gives more stable AuNPs, in which the particle size is controlled by adjusting the gold-to-citrate ratio. Their application in sensing is driven by their intrinsic property to emit a range of colors like red, brown, orange and purple in aqueous solutions as their size increases from 1 to 100 nm. Covalent binding and non-covalent binding (like electrostatic binding, hydrophobic binding *etc.*) are the major ways by which AuNPs bind with the substrate. Covalent conjugations involve the interaction of free molecules and thiolates on AuNP surfaces. Mie *et al.* found the relation between the diameter and the wavelength relation for AuNPs, which was found to be almost linear in nature.^202,203^

### 5.3 Lateral flow devices for the detection of sepsis biomarkers

The use of paper or other such porous materials provides capillary action that can be used to ensure the flow along a certain direction while ensuring their position and integrity. The porous nature of paper allows for the capillary action that draws the sample through the test strip. This movement is essential for the test to work, as it brings the sample into contact with the reagents that detect the target substance. The detection segments comprising the test line and control line are prepared on a thin strip while modifying its geometry and composition.^204–206^ Antibody–antigen conjugation is widely used in lateral flow devices. In lateral flow devices, test line and control line are formed by putting on the top layer and the porosity assisting *via* capillary action and ensure control on velocity by changing the porosity. In addition, the test line ensures the limit of detection by conjugation and confirms the presence of targeted biomarkers, and the control line ensures the specificity of the reaction, confirming that the test is working properly. The outcome of the test is assessed by the change in signal, like resistance and change in the color of the strip. Lately researchers have been using the change in visible range of EM (electromagnetic) wave spectrum have been assisted by mobile setup which takes picture of the spot of strip and calibrating intensity with quantity of conjugation reaction makes in quantifying the outcome.^207^ Lateral flow assay (LFA) combining immunolabeling and chromatography has gained increased attention in recent years.

Despite blood culture being the gold standard for sepsis detection, it is less desirable due to long incubation time, *i.e.*, 72 hours. Lateral flow devices were created which in many cases uses Latex nanoparticle, gold nanoparticle, silver nanoparticle, selenium nanoparticle *etc.* as an alternative method for sepsis detection at the point-of-care (POC) for binding with biomarker and higher visibility of test and control line. The nanoparticle properties like consistency in shape, size, morphology, and monodispersed are crucial in decreasing the assessment time, compared to the LOD (limit of detection) by varying size of nanoparticle od Gold and selenium. Here selenium and gold NPs were prepared by a chemical reduction method. The biomarker conjugated with nanoparticles is introduced and its antigen and antibody meet at the test line; at this point, the NP emits light in the visible range, and the intensity of test line gives an idea about antibody concentration. Based on visual inspection, the 40 nm AuNPs followed by the 150 nm SeNPs produced the greatest intensity test lines between 10 and 500 ng per mL IL-6, while the 310 nm SeNPs followed by 150 nm SeNPs provided the greatest intensity test lines at 0.1 ng per mL IL-6. It is proposed that steric hindrance constraints and the prozone effect are both responsible for the sensitivity decline that occurs with the increase in IL-6 concentration for bigger SeNPs, highlighting the significance of finding out which label size works best for a given LFD application.^208^

**Fig. 9:**
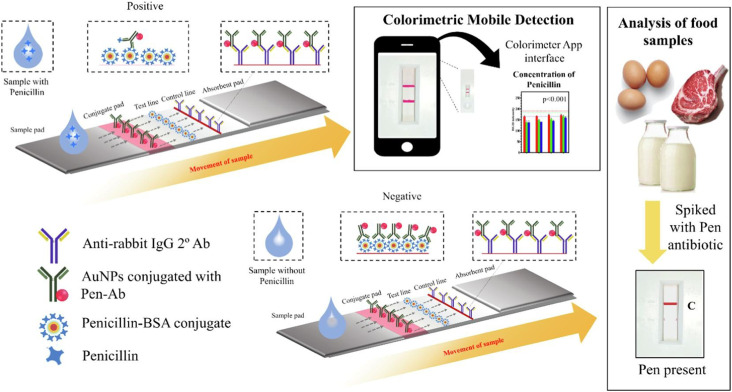
(i) Schematic of a colorimetric LFA system for detecting penicillin in food samples using AuNPs conjugated with penicillin antibodies. This competitive assay distinguishes positive (penicillin-present) and negative (penicillin-absent) food samples (shown in boxes) based on signal intensity, which is quantified using a mobile phone-based colorimetric application.^207^ (ii) Fluorescent LFA using various nanomaterials as reporters. (A) Visual detection of test results across different samples (NP: no penicillin; HB: high bovine; HL: high lamb; HP: high poultry). (B) Quantified fluorescence signal ratios across different reporter systems (CdSe/ZnS QDs, Europium chelate PS, and NaYF_4_-based upconversion nanoparticles). (C) Visual comparison between mock and blocked samples. (D) Corresponding quantitative fluorescence intensity ratios, highlighting significant signal suppression in blocked controls (**p < 0.01).^209^ (Reproduced with permission from Elsevier and the Royal Society of Chemistry, respectively. Copyright 2017, Elsevier Ltd; Copyright 2019, the Royal Society of Chemistry.).

**Fig. 10:**
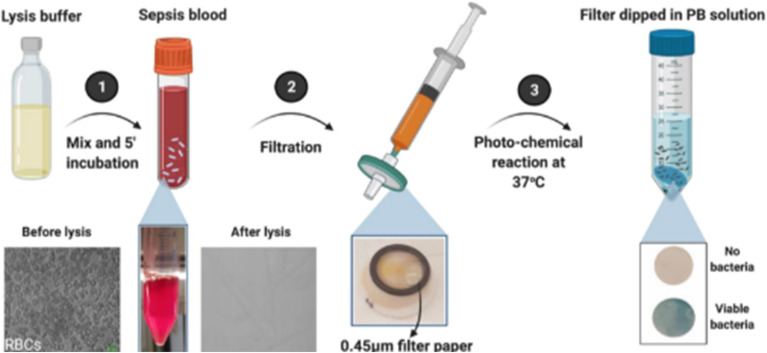
Schematic of the sensor workflow based on the paper's inherent capillary action, akin to a modified lateral flow assay (LFA): (1) selective isolation and detection of viable bacteria from sepsis blood. Sepsis blood is mixed with a lysis buffer and incubated for 5 minutes, enabling the selective lysis of red blood cells (RBCs), as visualized in the bottom-left panel (before and after lysis). (2) Lysed blood sample is passed through a 0.45 µm filter paper using a syringe, which captures intact bacteria while allowing lysed cell debris to pass through. (3) Bacteria-laden filter is subjected to a photo-chemical reaction at 37 °C and subsequently dipped into a phosphate-buffered (PB) solution containing chromogenic precursors. The presence of viable bacteria leads to a visible blue color on the filter paper, while filters without bacteria remain colorless. (Adapted with permission from Elsevier, *Biosensors and Bioelectronics*, 2021. Copyright 2021, Elsevier B.V.).^210^

**Fig. 11:**
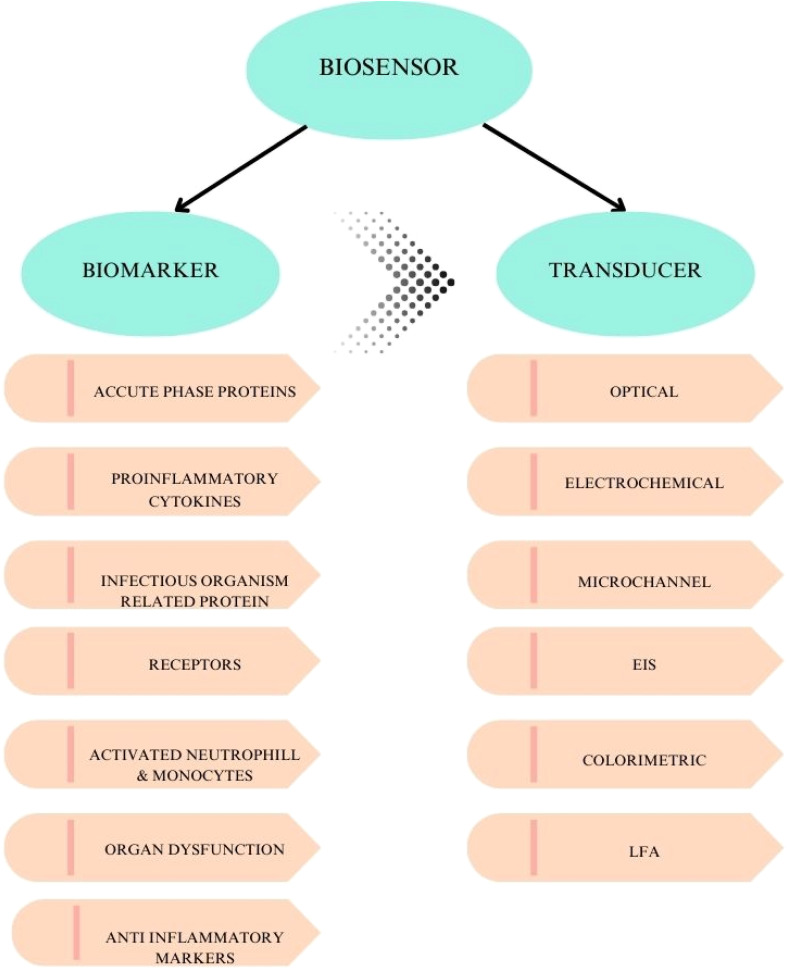
Schematic of the biomarkers and transducers used in sepsis detection, with their broad classification. (Adapted from Bonini A. *et al.*,^155^ “Emerging biosensing technologies towards early sepsis detection,” *Biosensors*, 2022, **12**, 894. Copyright 2022, MDPI, licensed under CC BY 4.0).

The ideal all-around label for IL-6 detection is thought to be the 150 nm-sized SeNP, which has the lowest LOD of 0.1 ng mL^−1^ and visual detection comparable to 40 nm AuNPs at high analyte concentrations. As 150 nm SeNPs have a lower limit of detection (LOD) than that of 40 nm AuNPs, they are particularly preferred in situations where prompt targeted medication delivery depends on early sickness identification, such as the early detection of sepsis.^208^Fig. 9 and 10 show the mechanism of lateral flow assay, using antibody conjugation, and Fig. 11 shows the schematic of the biomarker and transducer.

Patiño and group also developed a method to detect IL-6 biomarkers, but the uniqueness here lies in the technique of assessing the whole blood sample and using the mobile phones to an LOD of 0.1 pg mL^−1^ with a confidence level of 99%, as the biomarker's concentration increases to 12.5 pg mL^−1^.^209^ The mobile application uses the density of the spot formed in real time, which is inherently dependent on a calibrated function of intensity of purple spot with respect to the concentration of biomarker IL-6 and its interacted output with gold nanoparticles (AuNPs). The extraction of biomarkers from whole blood and ensuring the viability of bacterial cells are important. Current methods of identifying culture-based bacteria take time and, in many cases, give false-positive results. Therefore, Narayana Iyengar *et al.* developed a protocol to simultaneously identify the viable bacteria from blood by cell lysis followed by filtration and then utilizing the drop on a filter paper and antibiotic susceptibility testing to determine the minimum inhibitory concentration (MIC) using two antibiotics (ampicillin and gentamicin).^211^

The conjugated AuNPs are found to be anti-inflammatory, and hence, Taratummarat and group found the AuNPs of size of 20–30 µm to be suitable for use adjuvants to antibiotics in a mouse model.^210^ The death (not viable) and lysis of Gram-negative bacteria release endotoxin, the endotoxin released by lipopolysaccharide (LPS) from outer membrane of Gram-negative bacteria, and its presence in blood stream and this causes disruption in aligned crystals. The range of LPS tested is 5 mg mL^−1^.

#### 5.3.1 Biosensors based on lateral flow assay (LFA)

The lateral flow assay uses the concepts of fluid mechanics to develop paper-based devices for application in environmental science, healthcare, food and agriculture. It uses the material properties of paper in order to provide a platform for analytes to interact easily so that the outcome can be deciphered seamlessly. The low production cost, easy handling and operation make it suitable for resource scary places. However, the outcomes generated *via* this method often take time, which is a concern.^212,213^ In healthcare, for this method, bodily fluids like sweat, blood, serum, and urine are used for diagnosis. Chunxio and group integrated a CMOS-based sensor system and paper microfluidics to find out the change in absorbance to quantify the sepsis metabolites, *i.e.*, glucose and lactate.^213^ Alba-Patino *et al.* used gold nanoparticles (AuNPs) covered with carboxylate or amine moieties, or polyvinylpyrrolidone (PVP), to bind the antibody on paper substrates. The limit of detection (LOD) was 0.1 pg mL^−1^ in 17 minutes.^209^ The spot formed was assessed using a mobile phone, and its intensity was correlated with the concentration of IL-6. Alekhmimi *et al.* used a paper-based colorimetric device for MMP-9 detection using peptide-magnetic particle conjugates in a mouse model.^214^ Owing to the suitability of IL-6 to be used both as a proinflammatory and anti-inflammatory cytokine, it was used as a biomarker by Huang *et al.* By employing europium nanoparticles (EuNPs) coupled with an antibody, lateral flow immunoassay (LFIA) delivered a linear range of 2–500 pg mL^−1^ and a good sensitivity of 0.37 pg mL^−1^.^215^ Faridi *et al.* have used viscoelastic flow to sort the varying size of cells in a Newtonian fluid; bacteria with a smaller size remain in the stream and get separated. They used a combination of 2 µm- and 5 µm-sized particles and obtained very high separation values of 95% and 93%, respectively.^216^ The particles experienced shear-induced lift force, wall-induced lift force and viscoelastic force, which facilitated *E*. *coli* bacteria separation.

Luminescence is a common phenomenon, which is also found in nature as bioluminescence, a special form of chemiluminescence associated with living organisms and reactions catalyzed by enzymes. Therefore, the use of AuNPs, SeNPs, *etc.*, is important for generating the outcome. The combination of luminescence with the lateral flow assay (LFA) can be a useful method in diagnosis, as several nanomaterials produce better luminescence when mixed with blood contents. Ji and group developed a near-infrared-to-near-infrared up conversion nanoparticle (UCNP) immunolabeled LFA for background-free chromatographic detection of sepsis biomarker procalcitonin (PCT) in clinical human plasma and the limit of detection was found to be 0.03 ng mL^−1^.^208^ Since the clinical relevent PCT has a range of 0.05 to 10 ng mL^−1^, the PCT level of higher than 0.5 ng mL^−1^ in serum may lead to inflammation. It covers smoothly having its range 0.03–50 ng mL^−1^. Immunolabeling is possible due to fluorescent nanoparticles like Quantum Dots (QDs) and europium(iii) chelate fluorescent microspheres as shown by Li and group for SARS-CoV-2.^217^ The fluorescent properties are possible due to the conversion of smaller wavelengths into longer wavelengths in the visible range. The control line and test line are used to detect PCT by conjugating PCT with anti-PCT-mAb-UCNP to form a complex assisted by capillary force, which can then be retained at the T line by the immobilized capture antibody using a sandwiched structure. Matrix metalloproteinase-9 is involved in the fibrotic process in denervated muscles after sciatic nerve trauma, and the recovery is used as a biomarker for sepsis detection by Alekhmimi *et al.* utilizing a colorimetry method in a blood sample.^214^ They used BAL (bronchoalveolar lavage) and blood as the sample fluid. The diagnostic time was found to be less than 1 hour in FIP mice post-challenge. The gold strips were placed on the device to which MMP-9 and a magnetic bead mixer were attached. Damodara *et al.* developed a single-step separation and concentration method for biomarker proteins using agarose-based isoelectric gates.^218^ It detects bovine serum albumin (BSA) at a concentration level of 1–5 mg mL^−1^ with a peak concentration of over 300 mg mL^−1^. Damodara *et al.* combined isoelectric gateways with barium-immobilized metal affinity trapping to detect vitamin K-dependent protein C as a biomarker.^218^ This method is cheap, easy to perform and independent from immunoassay. With a relativity of more than 0.98, this method can measure protein levels from 4.46 µg mL^−1^ to 1.96 µg mL^−1^. In this biosensor, Anodic reservoir and cathodic membrane, uses barium affinity for protein and bound them to agorase gel in cathodic membrane region. The reduction in the volume of blood used is given priority nowadays, Hassan *et al.* used only 10 µL of whole blood for a point-of-care (PoC) device by detecting the nCD64 levels.^219^ In this method, they have used differential immunoaffinity capture technology to electrically quantify the antigen expression level on the CD64+ cells by selectively capturing using a biochip. The fluorescence was used to detect the CD64 levels around micropillars. Flow cytometry has been used to identify degranulation, but this is a tedious process requiring a large setup, and hence, Santopolo and group used plasmonic sensors with gold nanoparticles (AuNPs) to identify sepsis-derived hyperregulation.^220^ In this work, the degranulated and nongranulated neutrophils were differentiated using cationic proteins. This group found out that measuring degranulation is a better option than procalcitonin (PCT). Hou *et al.* used the margination property to separate *E. coli* and *Saccharomyces cerevisiae* spiked in a blood sample by passing them through a narrow channel of 20 × 20 µm^2^, and these bacteria are one of the reasons of sepsis.^220^ The smaller dimensions of the channel lead to the axial migration of RBCs (red blood cells) due to their deforming nature, while the particles like bacteria and platelets accumulate and move along the sidewalls of the channel. Therefore, they created 6 parallel microchannels at a flow rate of 6 mL per hour. The flow was assessed by confocal microscopy. Tanak *et al.* used a DETEecT sensor with a correlation value of >0.97 and a volume of blood of <40 µL in around 5 minutes to detect cytokines (IL-6, IL-8, and IL-10), chemokines (TRAIL and IP-10), and inflammatory biomarkers (PCT and CRP).^221^ Guo *et al.* used a Cu-BHT-based thin film for detecting the PCT levels, the anti-PCT antibody is covalently bonded to material surface, and it is measured by EIS. The LOD is 14.579 × 10^−9^ µg mL^−1^ and the linear detection range is 10^−7^ µg mL^−1^ to 0.1 µg mL^−1^.^222^

Nanoparticle imaging techniques for diagnosing sepsis: Imaging techniques for diagnosing and monitoring sepsis can be useful as they ensure the dynamic change in organs with time and present the growth of infection. Techniques like MRI (magnetic resonance imaging) image the internal organs and tissues of body using magnetic fields and radio waves.^223^ Ai *et al.* presents the use of superparamagnetic iron oxide nanoparticles (SPIONs) as contrast agents for detecting LPS or microbial cell wall components by binding with antibodies. Radioactive tracers are used in positron emission tomography (PET) for nuclear imaging for metabolic process visualization.^223^ Ai *et al.* showed the use of radiolabeled NPs, such as iron oxide nanoparticles or AuNPs, as contrasting agents in PET.^224^ Radio-labelled iron oxide is used to detect neutrophils by PET in LPS-induced lung injury.^223–226^

### 5.4 Electrochemical impedance spectroscopy (EIS)

**Fig. 12:**
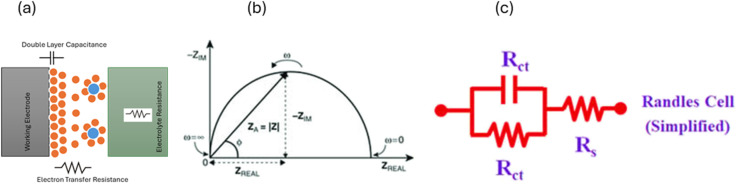
Schematic of (a) electrode–electrolyte interface showing charge transfer resistance and double-layer capacitance, (b) Nyquist plot, and (c) simplified Randles equivalent circuit model with solution resistance (*R*_s_), charge transfer resistance (*R*_ct_), and double-layer capacitance. (Adapted from ref. 222 with permission from John Wiley & Sons, Copyright 2005, Wiley-VCH).^227^

In electrochemical impedance spectroscopy (EIS), this technique can be used to assess the behavior of the surface electrode by applying sinusoidal potential and recording the current with the lowest harmful effect on the surface or polarization effects. A low voltage is kept in order to ensure the integrity of biological specimen whose impedance has to be measured. EIS has the ability to study the intrinsic material properties or specific processes that could influence the conductance, resistance, or capacitance of an electrochemical system. The impedance differs from resistance, since the resistance observed in DC circuits obeys Ohm's law directly. *R*_ct_ is the charge transfer resistance, *R*_s_ is the electrolyte resistance, and *C*_dl_ is the capacitance double layer,^227^ as shown in Fig. 12, and Table 13 lists the biosensors based on it. The Nyquist plot shows the relationship between the real and imaginary parts of impedance for different frequency ranges. Such plots are very helpful in biosensing, as they are unique for each frequency value. For a given frequency *f*, the angular frequency, current and impedance can be given as, here applied voltage is *E*^−^*w* = 2π*f**I* = *I* sin(*wt* + *Ω*)
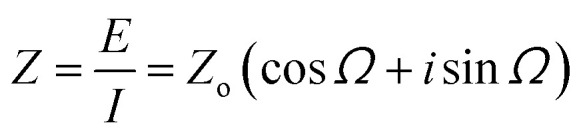


**Table 13:** Table of optical biomarkers and detection parameters

Substrate	Sample	Biorecognition element	Biomarker	Technique	LOD	Working range	Response time	Reference
Optic fiber	PBS buffer	Antibody	CRP	SPR	1.17 µg mL^−1^	0.01–20 µg mL^−1^	NA	228
Optic fiber	Human serum	Antibody	PCT	LSPR	95 fg mL^−1^	1–100 ng mL^−1^	<15 min	229
AuNPs	Mixed protein solution	Aptamers	IL-6	LSPR	1.95 µg mL^−1^	3.3–125 µg mL^−1^	5 min	230
Gold nanohole array (Au-NHA)	Spiked PBS sample	Antibody	CRP	Interferometry	18 mg mL^−1^	0–250 µg mL^−1^	1 min after sample incubation	231
	NA	Antibody	IL-6		88 mg mL^−1^	0–400 µg mL^−1^		
	NA	DNA capture probe	miRNA-16		6 mg mL^−1^	0.8–12.5 µg mL^−1^		
AuNPs	Broth culture	Electrostatic	Urease	LSPR	0.8 µg mL^−1^	0.8–12.5 µg mL^−1^	40 min	232
AgNPs-BP	Human serum	Aptamers	CRP	SERS	100 fg mL^−1^	10^−4^–10 ng mL^−1^	NA	233
	NA		IL-6		0.1 fg mL^−1^	10^−7^–10^−2^ ng mL^−1^		
	NA		PCT		1.0 fg mL^−1^	10^−6^–10^−1^ ng mL^−1^		
	Whole blood	Photocatalysis	*S. capitis*	Colorimetry	10^3^ CFU mL^−1^	10^2^–10^8^ CFU mL^−1^	<5 h	210
	NA		*E. coli*					
AgMNPs/CPs	Sterile human serum	Label-free	IL-3	SERS	1000 fM	1 pM–100 nM	Real time	234
	NA		PCT		100 fM	100 fM–100 nM		
Silicon chip	Human plasma	Antibody	CRP	WLRS	1 ng mL^−1^	0.05–200 µg mL^−1^	12 min	43

This technique has attracted attention due to its fast response, low detection limit (LOD), and low cost, and for its use in the real-time monitoring of samples instead of more traditional methods such as ELISA. One of the most important and practical advantages of impedimetric methods is that no enzymatic labels are needed to detect the samples. However, researchers are currently using conjugation methods on the electrode surfaces, which in turn change the impedance across the varying voltage source.^227,235^ The EIS technique can monitor the behavior of the surface electrode by applying sinusoidal potential and recording the current with the lowest harmful effect on the surface or polarization effects.

The use of nanomaterial has also increased in impedimetric biosensors, and the large surface area offers great receptor analyte interaction capability in a smaller area.^236^ Carbon-based nanomaterials and metal and metal oxide nanostructures like ZnO, CuO, NiO, TiO_2_, Fe_3_O_4,_ Au, Pt, Ag, and Pd were exploited for electrode modification due to their good biocompatible properties and inertness against oxidation reactions occurring on their surface, which make them suitable for various applications.^236^ Identifying the biomarkers in real time is crucial and necessary; Russell *et al.* developed a real-time IL-6 biomarker concentration label-free detector using a microelectrode based on electrochemical impedance spectroscopy (EIS), and the incubation period was found to be 25 minutes.^236^ In this work, eight microelectrodes of *r* = 25 µm placed in order were fabricated on a needle-shaped silicon substrate. This work also presented a contrasting view of decrement in impedance as the antigen binds the microelectrodes. Graphene is currently widely used in nanotechnology applications, due to its high capturing nature and smaller thickness. Electrochemical sensors (EC) are becoming valuable in point-of-care (POC) devices. Zupancic *et al.* used graphene for the detection of three biomarkers simultaneously, *i.e.*, PCT, CRP, and pathogen-associated molecular pattern. Their results show higher correlation with ELISA (*r*^2^ = 0.95).^237^ Highly oriented pyrolytic graphite (HOPG) has been used by Sharma *et al.* as an antibody-based label-free impedance biosensor.^139^ Kumar *et al.* aimed at developing a solid-state working electrode and improving the adhesion of nanomaterials at the electrode interface.^42^ A cerium oxide nanofiber (CeNF) developed *via* electrospinning was placed on the surface of a glassy carbon electrode (GCE) and employed for detecting TNF-α. Moreover, the effect of Nafion on interfacial activity was studied by comparative analysis between the electrochemical impedance spectroscopic (EIS) results of GCE/CeNF and the GCE/CeNF/Nafion.^238^

### 5.5 Optical method

**Fig. 13:**
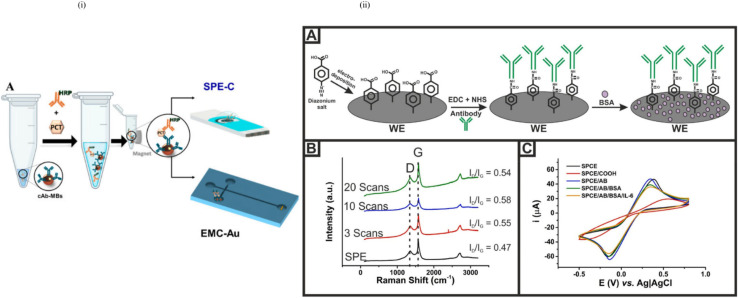
(i) (A) Schematic of the immunomagnetic separation and electrochemical detection process. HRP-labeled detection antibodies (dAbs) bind to the target analyte, forming a sandwich complex on capture antibody-coated magnetic beads (cAb-MBs). After magnetic separation and washing, the immunocomplex is deposited onto a screen-printed carbon electrode modified with gold (SPE-C, EMC-Au) for detection.^239^ (ii) Functionalization and characterization of the SPE-C electrode: (A) Stepwise surface functionalization of the working electrode (WE) *via* the EDC/NHS chemistry for antibody immobilization, followed by BSA blocking. (B) Raman spectra showing the increasing intensity and *I*_D_/*I*_G_ ratios with functionalization steps, confirming successful modification. (C) Cyclic voltammetry of the electrode at different modification stages, indicating changes in the electrochemical behavior due to surface chemistry alterations.^240^ (Adapted from Molinero-Fernández *et al.*, 2020 with permission from Elsevier, from Molinero-Fernández Á., Moreno-Guzmán M., *et al.* “Electrochemical immunosensors combined with immunomagnetic separation for sensitive biomarker detection.” *Biosensors and Bioelectronics*, 2020. Copyright © 2020 Elsevier B.V.).

Optical biosensors are the most commonly reported class of biosensors. The detection typically relies on an enzyme system that catalytically converts analytes into products that can be oxidized or reduced at a working electrode, maintained at a specific potential. The main advantage of this optical transducer is the low cost and the use of biodegradable electrodes. An optical biosensor is a compact analytical device, having a biological sensing element, integrated or connected to an optical transducer system, as shown in Fig. 13. The detection of specific bindings of the analyte of interest to the complementary optical biorecognition element is immobilized on a suitable optical substrate. The main biological materials used in optical biosensor technology are the optocouple enzyme/substrate, antibody/antigen, and nucleic acid/complementary sequences. In addition, microorganisms, animal or plant whole cells and tissue slices can be incorporated in the biosensing system. Recent advances and developments in molecular optoelectronics offer an alternative approach involving the use of optical biometric recognition systems.

Optical biosensors measure the variation in optical property (*e.g.*, chemiluminescence, absorbance, and fluorescence) triggered by the biorecognition reactions, where the output is directly proportional to the concentration of analytes.^241^ Two configurations of optical biosensors have gained great momentum for diagnosing infectious diseases. The first configuration is the lateral flow test.^242^ Optical methods have higher sensitivity than many diagnostic methods. Rascher *et al.* aimed at detecting an early-stage biomarker for sepsis detection using a total internal reflection-based point-of-care device (POCD), which also decreases the assay time, *i.e.*, less than 9 minutes.^242^ Fluorescence labeled detection antibody present along the path of flow, the incident light has an amplitude of 636 nm, while detected wavelengths are 650–655 nm were used for detection.^243^ Using label-free immunosensors for the determination of C-reactive protein (CRP) in human whole blood, with a total assay time of 12 minutes, white light reflectance spectroscopy was performed by Koukouvinos and group.^243^ The assay stage has three steps: 5-min reactions with the diluted whole blood samples, 3-min reactions with the biotinylated anti-CRP antibody, and 4-min reactions with streptavidin solution. The limit of detection (LOD) for CRP was found to be in the range of 400 µg L^−1^ to 50 mg L^−1^. Sharma *et al.* fabricated an optical biosensor (OB) for detecting severe infection. The sensor integrated information from heart rate, pulse oximetry, kidney function, NO level, vascular diameter and inflammation.^244^ CRP was detected by Sridevi and group by using an optical fiber device, within 15 minutes.^121^ A photonic biosensor has been developed by Fabri-Faja *et al.* to detect multiple biomarkers, *i.e.*, C-reactive protein (CRP) and interleukin-6 (IL6), and miRNAs with LODs of 18 mg mL^−1^, 88 mg mL^−1^ and 1 mM (6 mg mL^−1^), respectively.^230^

Fluorescent probes are key elements in the biosensing and diagnostics platform. Combined with efficient imaging, it can help in visualizing the biomolecules of interest, and real-time analysis can be performed. Fluorescent compounds bind (sometime interact with weak bonds like hydrogen bond) with a specific compound available in the biomolecule. Bauer *et al.* used a pH-sensitive probe with FRET (Förster resonance energy transfer) for sepsis detection.^245^ Feng *etal.* used polymyxin B derivatives for detecting lipopolysaccharides (LPS) on Gram-negative bacteria.^246^ Hoffmann *et al.* used a flow cytometry-based method for CD64-FITC detection.^247^ Accardo *et al.* used “all-in-one” probes to simultaneously identify pathogens and assess antibiotic effectiveness.^248^ Small-molecule organic probes, such as reactive oxygen species-responsive dyes, were used for intracellular identification of inflammatory markers. Fluorescent protein probes, including GFP-tagged antibodies, were used for the real-time monitoring of immune cell migration in sepsis models. Quantum dot probes exhibit high photostability for the swift identification of sepsis-inducing pathogens, such as *Klebsiella pneumoniae*. Near infrared emitting nanoparticle probes, like NIR-II nanoparticles, for imaging dysfunction in sublingual microcirculation during septic shock. Yang *et al.* used a DLF-1 probe to quantitate Mtb, which was successfully applied to identify genes critical for cell invasion.^249^ Bhuin *et al.* developed single but simple organic molecules possessing numerous realistic photophysical properties such as mechanofluorochromism, aggregation-induced emission (AIE) properties, solvatochromic DSE-gens (dual-state emissive fluorogens), and viscofluorochromism,^250^ they also developed ingle 2,3,4-trimethoxybenzene-linked DSE-gen (the lead) for bioimaging efficacy and a way to detect dead cancer cells selectively. Zhang *et al.* have discussed different methods for the real-time detection of cancer using targeted fluorophores.^251^ All these works show the selective and improved ways of fluorophore applications.

The rapid detection and identification of bacteria directly from the whole unprocessed blood sample is known as an optical method of detection. Inexpensive optical biosensors are becoming important nowadays. Although such optical techniques were initially devised to study cancerous changes in epithelial tissue by characterizing the change in wavelength when light waves interact with tissue. In the beginning, Wyatt demonstrated different angular dependence of scattering for three bacterial strains.^252^ The time-dependent fluorescence and Raman spectroscopy were employed by Layne, Bigio and colleagues for the detection of bacteria.^252^ A. Katz, *et al.,* demonstrated Fourier-transform infrared spectroscopy (FTIR) of the *Escherichia coli* bacterial strain.^253^ Konokhova *et al.* used optical methods to find the concentration of viable bacteria.^254^ Banada *et al.*, used scattering to identify individual strains of different size bacteria, similarly angle resolved scattering combines width flow cytometry to identify strain of *E coli* by scanning flow cytometry.^255^ Angular imaging patterns of a forward-scattering laser beam were also employed for the detection of bacterial pathogens in food.^256^ Qiu *et al.* used a novel optical spectroscopic method for the rapid detection and identification of bacteria directly from whole blood, using light scattering spectroscopy (LSS)-based techniques.^256^ The accuracy of this method was found to be 10 nm, with testing done for bacteria in water suspension and in blood, and eventually, several typical Gram-negative (*E. coli*, *K. pneumonia*, and *P. aeruginosa*) and Gram-positive (*S. aureus*) strains of bacteria, known to cause sepsis, were identified with LSS. Damodara *et al.* captured and quantified cfDNA (cell free DNA) as a sepsis biomarker present in plasma,^257^ the device was tested to find buffered in the range of 1–6 µg mL^−1^, sensitivity of 5.72 AU µg^−1^ mL^−1^. For this process a silicone cartridge, with the assistance of fluorescence dye, the imaging is done for measurement. The point-of-care (POC) devices have limitations of low sensitivity, limited multiplexing capability, and low throughput. Chin *et al.* developed a multiple biomarker detection POC device, utilizing a portable imaging system, the amplification is done through chemical and plasmonic methods for det a portable imaging system in about an hour.^258^ Battaglia *et al.* developed a molecularly imprinted polymer (MIP)-based surface plasmon resonance (SPR) biosensor, with LOD values of 15 ng mL^−1^ and 30 ng mL^−1^, respectively, for equine and canine PCT.^259^ The use of two-dimensional chromatography for the *in situ* detection of multiple sepsis biomarkers, *i.e.*, CRP, PCT and lactate, in the same device has become a viable alternative. Kemmler *et al.* developed an integrated multiparameter on-chip immunofluorescence assay to detect CRP IL-6, PCT, and NPT, by measuring the total internal reflection fluorescence (TIRF).^260^ Early detection of CitH3 (citrullinated histone H3) can prevent septic shock. Y. Park, *et al.* developed a 2.5 × 2.5 mm^2^ plasmo-photoelectronic nanostructure device for CitH3 detection in the range of 10^−4^ to 0.1 ng mL^−1^ within 20 minutes. Preventing septic shock by early detection is crucial, and neutrophils are important cells for ensuring the body defense.^261^

### 5.6 Electrochemical method

Electrochemical methods monitor the reaction kinetics of an electroactive species at the electrode/solution interface by measuring the current, voltage, or impedance, as recently reviewed by others.^262^ Micro-structured electrochemical sensors can be manufactured using well-established methods for microchip fabrication.^263,264^ This makes it easy to fabricate sensor arrays, wearable devices, and ultramicroelectrodes integrated in microfluidic platforms for point-of-care devices. Exosomal markers are extracellular vesicles secreted by cells, representing their parent cell. It may be proteins, lipids and nucleic acids like miRNAs. Like miR-150 is expressed in mature lymphocytes responsible for sepsis, cancer and mycordial injury diagnosis. Similarly, MiR-223 present in myeloid lineage cells regulates neutrophil activation.^265^ Ondevilla *et al.* developed an electrochemical biosensor for tumor necrosis factor-alpha (TNF-α), interleukin-6 (IL-6), and microRNA-155 (miR-155) in a lipopolysaccharide (LPS)-induced septic mouse model, with limit of detection (LOD) values of 0.84, 0.18, and 0.0014 pg mL^−1^, respectively.^265^ Min *et al.* developed a magneto electrochemical sensor integrated with a mobile to detect IL-3.^266^ The time taken in detection was less than 1 hour, with an LOD less than 10 pg mL^−1^. Here magnetic beads provide a large surface area for target capturing. Without the need for diluting or sample processing for whole blood, Tanak *et al.* detected 8 biomarkers (IL-6, IL-8, IL-10, IP-10, TRAIL, d-dimer, CRP, and G-CSF) simultaneously within 5 minutes with around 100 µL of blood sample. The DETecT sepsis (Direct Electrochemical Technique Targeting Sepsis) 2.0 sensor measured the binding at metal–semiconductor interfaces.^267^ Lu *et al.* developed a dual-channel electrochemical sensor for detection in the range of 0.5–1000 pg mL^−1^ for lipopolysaccharide (LPS) and 0.1–20 µg mL^−1^ for C-reactive protein (CRP), and the results were consistent with the ELISA results.^268^ Mansor *et al.* developed electrochemical sensors for bacterial sepsis infection which use the production of hydrogen peroxide, which presence was indicated by the redox characteristics of potassium ferricyanide, K_3_Fe(CN)_6_.^269^ In addition, hydrogen peroxide was generated by the reaction of sPLA2-IIA with its substrate. The linear range is 0.01–100 ng mL^−1^, with a limit of detection of 0.005 ng mL^−1^. Tian *et al.* developed an electrochemical sensor with better stability and higher accuracy to detect fibronectin (FN) in serum in the range of 15.625–500 ng mL^−1^.^270^ Kaur *et al.* used cyclic voltammetry (CV), developed an electrochemical sensor and found that the charge for LPS-absent bacteria is more than that for LPS-containing bacteria. The samples were encapsulated in a micro-scaffold to assess the change in pH in medium due to redox processes.^271^ Lactate detection in severely ill sepsis patients is a crucial biomarker, Thongkhao *et al.* developed an electrochemical sensor using polyurethane–polyaniline–*m*-phenylenediamine arranged layer by layer, with a linear range of 0.2–5 mmol L^−1^, and the outcomes of the sensor are highly closer to those of the enzymatic colorimetric gold standard method (*p* > 0.05).^272^ Kiatamornrak prepared a TiO_2_ sol-G nanocomposite and coated it onto a screen-printed carbon electrode (SPCE) to detect non-immobilized lactate oxidase (LOx).^273^ Chen *et al.* prepared a zeolite- and iron oxide-complexed capacitance electrode on which anti-interleukin-3 (anti-IL-3) is present, so that *via* amine linking IL-3 gets attached.^274^ Li *et al.* developed a wearable and battery-free wound dressing system for wireless and early sepsis diagnosis by the real-time detection of PCT.^275^ Fernandez *et al.* used PCT as a protein biomarker, for developing two ways of PT immunoassay detection.^276^ In first disposable screen-printed carbon electrodes (SPE-C, on-drop detection) and electro-kinetically driven microfluidic chips with integrated Au electrodes (EMC-Au, on-chip detection). Both approaches exhibited enough sensitivity (limit of detection (LOD) values of 0.1 and 0.04 ng mL^−1^ for SPE-C and EMC-Au, respectively; cutoff = 0.5 ng mL^−1^), an adequate working range for the clinically relevant concentrations (0.5–1000 and 0.1–20 ng mL^−1^ for SPE-C and EMC-Au, respectively), and good precision (RSD < 9%), using low sample volumes (25 µL) with total assay times less than 20 min. Fig. 13 shows the schematic of electrochemical detection and association characterization, and Table 14 shows the associated biosensors.

**Table 14:** Biomarkers detected by electrochemical methods and crucial detection parameters

Electrode	Sample	Biomarker	Biorecognition element	Technique	LOD	Working range	Response time	Reference
Gold	Buffer	PCT	BP3 peptide	EIS	12.5 ng mL^−1^	0.013–0.25 µg mL^−1^	NA	277
Carbon screen printed	Human serum	PCT	Antibody	Amperometric	0.1 ng mL^−1^	0.5–1000 ng mL^−1^	<20 min	239
Gold	Plasma	PCT	Antibody	Amperometric	0.04 ng mL^−1^	0.1–20 ng mL^−1^	<20 min	
Carbon screen printed	Plasma	CRP	Antibody	Amperometric	0.80 µg mL^−1^	2–100 µg mL^−1^	5 min	278
Carbon screen printed	Plasma	CRP	Antibody	Amperometric	0.058 µg mL^−1^	1–100 µg mL^−1^	5 min	279
Glassy carbon electrode	Diluted serum	PCT	Antibody	Amperometric	0.011 pg mL^−1^	0.0001–100 ng mL^−1^	50 min	280
Glassy carbon electrode	Diluted human serum	PCT	Antibody	DPV	0.46 pg mL^−1^	0.001–100 ng mL^−1^	NA	281
Glassy carbon electrode	Human serum	PCT	Antibody	DPV	0.3 pg mL^−1^	1 pg mL^−1^–100 ng mL^−1^	NA	282
Gold interdigitated electrode	Human serum	PCT, CRP	Antibody	EIS	10 ng mL^−1^	0.01–10 ng mL^−1^	<15 min	283
Gold electrode	Clinical sample	PCT, CRP	Antibody	Amperometric	10 ng mL^−1^	0.01–10 ng mL^−1^	<15 min	
Gold electrode on microneedle	Human serum spiked	IL-6	Antibody	DPV	NA	20–100 pg mL^−1^	3 min	274
Gold interdigitated	Human serum spiked	IL-3	Antibody	Capacitive	3.0 pg mL^−1^	3.0–100 pg mL^−1^	NA	284
Gold screen printed	Plasma/serum from clinical sample	IL-3	Antibody	Chronoamperometry	10 pg mL^−1^	10–10^4^ pg mL^−1^	<1 h	285
Gold	Plasma	IL-6, IL-8, IL-10, TRAIL, IP 10	Antibody	EIS	0.1, 0.1, 1.0, 1.0, 1.0 pg mL^−1^	0.01–10^4^, 0.1–5000, 0.1–10^3^, 1.0–2 × 10^3^ pg mL^−1^	5 min	286
Disposable sensor cartridge with a gold-based array electrode	Clinical samples	IL-6, IL-8, IL-10, TRAIL, IP 10	Antibody	Label-free non faradic impedance spectroscopy	0.1, 0.1, 1.0, 1.0, 1.0 pg mL^−1^	0.01–10^4^, 0.1–5000, 0.1–10^3^, 1.0–2 × 10^3^ pg mL^−1^	5 min	287
Gold	Human blood	16S RNA from *S. aureus*, *E coli*, *P aeruginosa*, *P. mirabilis*	RNA specific probe	Amperometry	290 CFU mL^−1^	NA	<1 h	288
Indium tin oxide coated glass	Spiked human urine	*K. pneumoniae*	Conductive MIP	DPV	1.35 CFU mL^−1^	1.0–1.0 × 10^5^ CFU mL^−1^	3 min	289
Gold	Buffer solution spiked clinical strains	DNA from *E. coli*, *S. aureus*	CRISPR/Cas12a	EIS	3.0 nM	3 –18 nM	1 h	290

The use of multiplex biosensors is becoming increasingly needful nowadays due to simultaneous detection of multiple biosensors to ensure identification and ensures of biomarkers simultaneously. Gao *et al.* developed a multiplex biosensor to detect the species-specific sequences of the 16S ribosomal RNA of bacteria for pathogen identification in physiological samples without preamplification.^240^ Crapnell & Banks developed functionalized in-house 3.1 mm-diameter screen-printed electrodes (SPEs) in conjunction with a thermal detection methodology for the detection of IL-6, without getting significant interference from PCT by only utilizing 110 µL cell volume.^291^ Graphene is known to adsorb single-stranded DNA due to noncovalent π–π bonds, but not double-stranded DNA. This approach does not require any surface functionalization and allows the detection of primer concentrations at the endpoint of reactions.^292^ As recently demonstrated, the crumpled gFET over the conventional flat gFET sensors due to their superior sensitivity is chosen. The end point of the amplification reaction was detected from initial concentration as low as 8 × 10^−21^ M in 90 minutes.

### 5.7 Microchannel-based methods

**Fig. 14:**
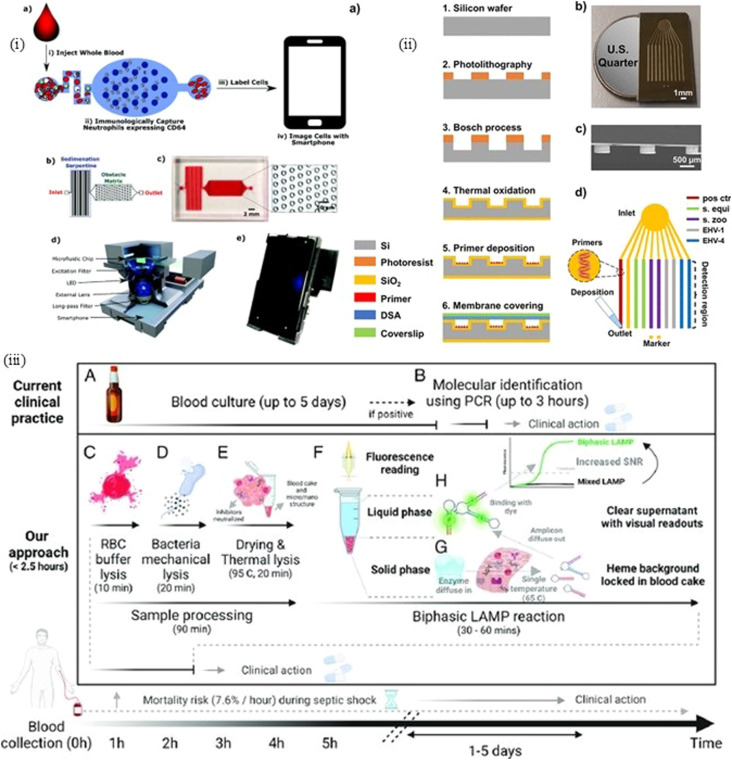
(i) Schematic of the device setup for immunological capture and smartphone-based imaging of neutrophils expressing CD64. (a) Whole blood is injected into the microfluidic chip. Neutrophils are captured using surface markers, labeled, and then imaged *via* a smartphone. (b and c) Microfluidic designs for cell separation using inertial and sedimentation-based methods. (d) Integrated optical detection setup using a smartphone and excitation/emission filters. (e) Portable diagnostic device capturing fluorescent signals from labeled cells.^297^ (ii) Fabrication workflow of a silicon-based microfluidic chip for nucleic acid testing: (a) Steps from silicon wafer photolithography to membrane sealing. (b) Fabricated chip compared to a US quarter. (c) Cross-sectional view showing channel dimensions. (d) Schematic of primer deposition and amplification zones.^19^ (iii) Comparison of current clinical practice (A and B) *versus* the proposed method (C–H) for blood-borne infection detection. Conventional workflow requires 1–5 days for blood culture and PCR-based identification. The novel approach completes diagnosis within ∼2.5 hours by integrating rapid red blood cell and bacterial lysis (C–E), thermal and mechanical lysis (F), and biphasic LAMP reaction (G and H) that separates solid and liquid phases for enhanced signal-to-noise ratio and visual readouts.^298^ (Reproduced with permission from Elsevier and the Royal Society of Chemistry. Copyright 2017, Royal Society of Chemistry; Copyright 2020, Elsevier B.V.).

**Fig. 15:**
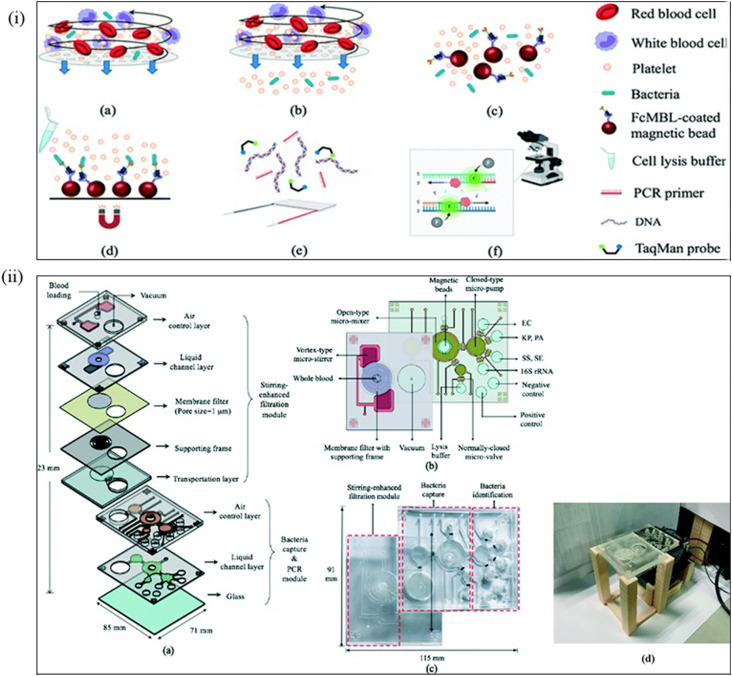
(i) (a–c) Schematic of a blood sample processing using FeC-MBL-coated magnetic beads for selective bacterial capture from whole blood by applying a rotating magnetic field to enhance bead-bacteria interactions. (d) Post-capture magnetic separation isolates bacteria-bound beads while removing blood components. (e) Captured bacteria are lysed, releasing DNA for amplification using PCR primers and TaqMan probes. (f) Optical readout of the amplified DNA using a detection system. (ii) Detailed view of the multilayered microfluidic cartridge comprising layers for fluid handling, filtration, lysis, magnetic actuation, and nucleic acid amplification. Zoomed-in schematic of the detection chamber layout and valve mechanisms. Bright-field microscopic images confirming bacterial capture. Portable detection system showing the final prototype, designed for point-of-care diagnostics.^293^ (Reproduced from Kang J., *et al.* “Integrated immunomagnetic microfluidic system for rapid and sensitive bacterial detection from whole blood.” *Biosensors and Bioelectronics*, 2019, with permission from Elsevier. Copyright 2019, Elsevier B.V).

**Fig. 16:**
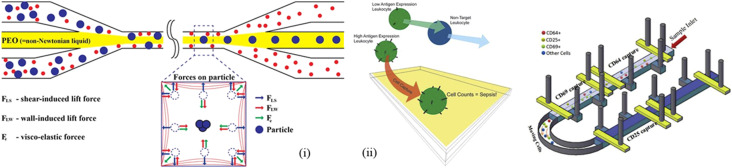
(i) Illustration of the microfluidic techniques for leukocyte separation and sepsis diagnosis, (i) inertial focusing using viscoelastic PEO (polyethylene oxide) fluid enables particle alignment due to shear-induced, wall-induced, and viscoelastic lift forces.^300^ (ii) Conceptual framework for identifying high-antigen-expressing leukocytes as indicators of sepsis. (Right) Microfluidic chip design employing antibody-coated pillars for label-specific separation of leukocyte subtypes (*e.g.*, CD64+, CD25+, and CD69+).^301^ (Reproduced from the Royal Society of Chemistry, Copyright 2017 and Copyright 2019, the Royal Society of Chemistry.).

Bodily fluid and other fluidic components are used in the detection and quantification of biomarkers or bacterial loads. The combination of fluid mechanics and components size at very small-scale work of a different nature. Ensuring the flow of pathogen-loaded blood in a constraint pathway can help in identification. Therefore, the microfluidic method is one of the methods developed to utilize fluid mechanics characteristics and ensure the flow in micro-scaled channels, allowing them to interact with different systems like optical, resistive, and potentiometric, and then read out the results according to the requirement. Culture negative results for bacteria causing sepsis occurs when bacteria agent can't be identified. This leads to extended duration for the intake of empiric antibiotics. Therefore, Zhou and group developed a microfluidic platform for capturing CD64, CD69, and CD25 expression with excellent accuracy.^290^ They found that CD64 and CD69 cell separation gives strong assay. Fang and group have developed a membrane-based integrated microfluid chip in which DNA is amplified by PCR and assessed by fluorescence to detect Gram-positive and Gram-negative bacteria.^293^ This device separated all white cells and 99.5% red cells from bacteria. However, here time taken in this detection process was very high, around 4 hours. Blood stream infection (BSI) is critical, and infection needs to be identified and removed quickly. The use of mild detergent solution and deionized (DI) water in microfluidic channels can be proved to be helpful. Zelenin *et al.* prepared a microfluidic channel with three inlets and introduced detergent, which can lyse most blood cells and DI water removes all blood cells, showing 100% bacteria recovery.^293^ Kundu and group integrated surface plasmon resonance (SPR) with microfluidics for easy readout of biomarker, in which analytes as assessed at junction to detect PCT with sensitivity 0.0643 a.u. pg^−1^ ml and LOD 0.0224 a.u. pg^−1^ ml.^294^ At the onset of sepsis infection when infection level is low, there are chances of their phagocytoses by immune cells. Hence, Liao *et al.* used a deformability test and microscopic imaging to show the presence of intracellular bacteria in phagocytic blood cells.^295^ In addition, they developed a microfluidic biosensor to passively sort, concentrate and quantify rare monocytes with internalized pathogens (MIP) from uninfected monocyte populations for phagocytosis detection within 1.5 hours. Ganguli *et al.* reported a biphasic approach to reduce the time of detection and increased molecular sensitivity along with reducing the time of detection, *i.e.*, less than 2.5 hours as compared to blood culture followed by PCR, which, despite being gold standard, takes 5 days to give a negative result.^296^ In this study they have taken 3 bacterial and one fungal species from around 1 mL of blood, to present the single molecule sensitivity using Loop mediated isothermal amplification (LAMP) to detect Gram-positive methicillin-resistant and methicillin-susceptible *Staphylococcus aureus* bacteria, Gram-negative *Escherichia coli* bacteria, and *Candida albicans* (fungus) from whole blood with a limit of detection (LOD) of 1.2 colony-forming units (CFU) mL^−1^ from 0.8 to 1 mL of starting blood volume. CD64 is a crucial biomarker in detecting sepsis; although no single or even a combination of biomarkers has been validated for the diagnosis of sepsis, multiple studies have shown the high specificity of CD64 expression on neutrophils (nCD64) to sepsis.^297^ Currently, flow cytometry is used for measuring the level of CD64, but its manual sample preparation and long incubation times are not desirable. Therefore, Ghonge *et al.* used a smartphone-imaged microfluidic biochip for detecting nCD64 expression within 50 min using unprocessed blood by capturing the nCD64 along a staggered array of pillar, which have been functionalized with the antibody of CD64, and the fitting of a newly developed method shows strong correlation with flow cytometry (*R*^2^ = 0.82).^298^ The level of CD64 is crucial and important in deciding the beginning of sepsis infection; it strongly correlates with sepsis. Hassan *et al.* used a very small volume of blood sample *i.e.*, 10 µL, and recorded the level of nCD64 during stay in hospital.^297^ Thus, the work is inspired by Coulter method of cell counting, where the CD64 is captured using its antibody on neutrophil's membrane surface. Fang *et al.* developed an integrated microfluidic chip (IMC), consisting of a membrane based filtration separating in 90 minutes, is then followed by miniature PCR setup for bacteria identification, taking around 4 hours. The limit of detection is 5 CFU per reaction.^299^Fig. 14 shows the setup of microfluidic channel consisting of serpentine and micropillar-based array to capture CD64. Fig. 15 shows the steps involved in the fabrication of microfluidic channels using photoresist. Fig. 16 shows the principle involved in the biphasic reaction for pathogen trapping and their DNA amplification (Table 15).

**Table 15:** Table of the fluorescence methods for sepsis detection and crucial detection parameters

Technique	Sample	Flow delivery	WR	LOD	Analysis time	Sample volume	Reference
Fluorescence	Serum	PPy/Ni/PtNPs	0.5–150 ng mL^−1^	0.07 ng mL^−1^	30 min	25 µL	302
Fluorescence	Serum	rGO/Ni/PtNPs	0.03–1280 ng mL^−1^	0.01 ng mL^−1^	5 min	25 µL	303
Fluorescence	Serum	rGO/Ni/PtNPs	0.01–128 ng mL^−1^	0.003 ng mL^−1^	15 min	2 µL	304
Colorimetry	Whole blood	Fe_2_O_3_	1–20 ng mL^−1^	0.56 ng mL^−1^	13 min	10 µL	305
Fluorescence	Serum	LFIA	1–300 µg mL^−1^	0.3 µg mL^−1^	>1 h	NA	306
Fluorescence	Serum	LFIA	0–1000 pM	42.5 nM	<35 min	50 µL	307
Fluorescence	Serum	LFS	50–250 µg mL^−1^	1 ng mL^−1^	<20 min	100 µL	308
Fluorescence	Serum diluted	Pressure	3.13–100 µg mL^−1^	1.87 µg mL^−1^	45 min	50 µL	307

The use of inertial focusing method is easy to use, and the low-cost microfluidic method is used for separating particles varying in size. Blood stream infection-causing pathogens can be separated by this method. Faridi *et al.* developed and fabricated an elasto-inertial microfluidic device for the continuous separation of 5 µm particles from 2 µm particles at a yield of 95% for 5 µm particle and 93% for 2 µm particles at respective outlets.^309^ Next, bacteria were continuously separated at an efficiency of 76% from the undiluted whole blood sample. By using the combination of elastic and inertial force, we obtained highly efficient particle separation. Similarly, Ohlsson used acoustophoresis with microfluidics for bacterial separation with *Pseudomonas putida* spiked into whole blood, revealing a detection limit of 1000 bacteria/mL for this first-generation analysis system.^309,310^

Zhou *etal.* fabricated a microfluidic channel for the capture and detection of CD64 and CD69 cells. To validate this assay, 40 sepsis patients and 10 healthy volunteers were enrolled in this study.^290^ Sepsis patients were divided into culture-positive (*n* = 12) and culture-negative (*n* = 21) cases. Capture, of CD64+ cell demonstrated excellent accuracy for sepsis detection, with an area under the receiver operating characteristic curves (AUC) of 0.962. Damodara *et al.* used cell-free DNA (cfDNA) present in the plasma of sepsis-infected blood stream. Patients in serious cases have higher correlation between the cfDNA concentration and the chances of survival; in recent works, they have found that the sepsis patients entering the intensive care unit who were likely to survive had a total cfDNA concentration of 1.16 ± 0.13 µg mL^−1^ compared to 4.65 ± 0.48 µg mL^−1^ of non-survivors.^257^ Here, they used a threaded silicone device for storing fluorescent dyes, which reduced the preparation time and detection cost. The device was demonstrated for use in the quantification of buffered cfDNA samples in a range 1–6 µg mL^−1^ with a sensitivity of 5.72 AU µg^−1^mL^−1^ and with cfDNA spiked in plasma with a range of 1–3 µg mL^−1^ and a sensitivity of 5.43 AU µg^−1^ mL^−1^.

One of the major disadvantages of detecting bacteria is the lack of sensitivity when we are trying to detect lower levels of bacteria. Moreover, the primers used in methods like PCR may interfere with inhibitory agents like hemoglobin, *etc.* So, Ganguli *et al.* developed a biphasic approach to ensure that we can approach to the Nucleic acid components like DNA rather than forcing them to come out of dried blood matrix. Therefore. in this method they dried whole blood, leading to the development of microporous channels, and eventually added primers to this solid blood cake to attach DNAs with primers. This has led to biosensor development with single-molecule sensitivity for 3 bacteria and 1 fungal species from ∼1 mL of blood in <2.5 h.^296^

Utilizing specific nucleic acid sequences for four equine respiratory pathogens as representative examples, we demonstrated the ability of the system to utilize a single 15 µL droplet of test sample to perform selective positive/negative determination of target sequences, including integrated experimental controls, in approximately 30 min.^311^ This approach utilizes loop-mediated isothermal amplification (LAMP) reagents predeposited into distinct lanes of the microfluidic chip, which, when exposed to target nucleic acid sequences from the test sample, generates fluorescent products that, when excited by appropriately selected light-emitting diodes (LEDs), are visualized and automatically analyzed by a software application running on the smartphone microprocessor. Choudhury *et al.* developed organic LED (OLED) for glucose biosensing, and these can be easily fabricated on a glass or plastic substrate, leading to a simple and compact device.^311^ Lian *et al.* used phycoerythrin (PE) for OLED-based SARS-CoV-2 antibody detection. Lian *et al.* developed an OLED-based fluorescence sensor to detect as low as 1 × 10^−9^ m of ssDNA-Cy5 in fetal bovine serum (FBS).^312^

A rapid and automated device to periodically measure nCD64 expression at the point of care (POC) could lead to timely medical intervention and reduced mortality rates.^297^ Current accepted technologies for measuring nCD64 expression, such as flow cytometry, require manual sample preparation and long incubation times. For POC applications, however, the technology should be able to measure nCD64 expression with little to no sample preparation. In this paper, we demonstrate a smartphone-imaged microfluidic biochip for detecting nCD64 expression within 50 min. In our assay, first unprocessed whole blood is injected into a capture chamber to immunologically capture nCD64 along a staggered array of pillars, which were previously functionalized with an antibody against CD64. Then, an image of the capture channel is taken using a smartphone-based microscope. This image is used to measure the cumulative fraction of captured cells (*γ*) as a function of length in the channel. During the image analysis, a statistical model is fitted to *γ* in order to extract the probability of capture of neutrophils per collision with a pillar (*ε*). The fitting shows a strong correlation with the nCD64 expression measured using flow cytometry (*R*^2^ = 0.82). Finally, the applicability of the device to sepsis was demonstrated by analyzing nCD64 from 8 patients (37 blood samples analyzed) along with the time they were admitted to the hospital. The results from this analysis, obtained using the smartphone-imaged microfluidic biochip, were compared with the flow cytometry results. Again, correlation coefficient *R*^2^ = 0.82 (slope = 0.99) was obtained, demonstrating a good linear correlation between the two techniques.

### 5.8 Machine learning (ML)-based approach

The onset of sepsis can also be recognized by the change in leukocyte count and CD64 level. Hassan *et al.* used this understanding to quantify sepsis using ANN (artificial neural networks), showing a higher accuracy.^313^ Taneja *et al.* studied the patient data from U.S. hospital and applied machine learning data on multiple biomarkers (IL-6, nCD64, IL-1ra, PCT, MCP1, and G-CSF) and combined EMR (electronic medical record) data achieved an area under the receiver operating characteristic (ROC) curve (AUC) of 0.81, while EMR data alone achieved an AUC of 0.75 for the early detection of sepsis.^314^ Taneja continued the above work and developed machine-learning model using this dataset outputs a score with not only diagnostic capability but also prognostic power with respect to hospital length of stay (LOS), 30-day mortality, and 3-day inpatient re-admission and obtained area under the receiver operating characteristic curve (AUROC) for diagnosis of sepsis was 0.83.^315^ Hassan *et al.* used computational methods using hierarchical clustering in artificial neural network (ANN) to find the rate of cell capture and showed increased accuracy, with the data for 106 patients.^297^ A single-center study, including a representative cohort of 325 infants (2866 hospitalization days), was used for sepsis prediction using the Naïve Bayes algorithm in a maximum *a posteriori* framework up to 24 h before clinical sepsis suspicion.^316^ This enabled a prediction of sepsis with an area under the receiver operating characteristic curve of 0.82, up to 24 h before clinical sepsis suspicion. Since wearables are very important in recording and understanding vital conditions, Gupta *et al.* used cuff-less blood pressure measurement with the PPG (photoplethysmography) signal.^316^ Giordano *et al.* used six vital sign predictions with tiny machine learning (TinyML) algorithms, enabling on-device real-time sepsis prediction for the development of SepAI.^317^

### 5.9 Aptameric biosensing

**Fig. 17:**
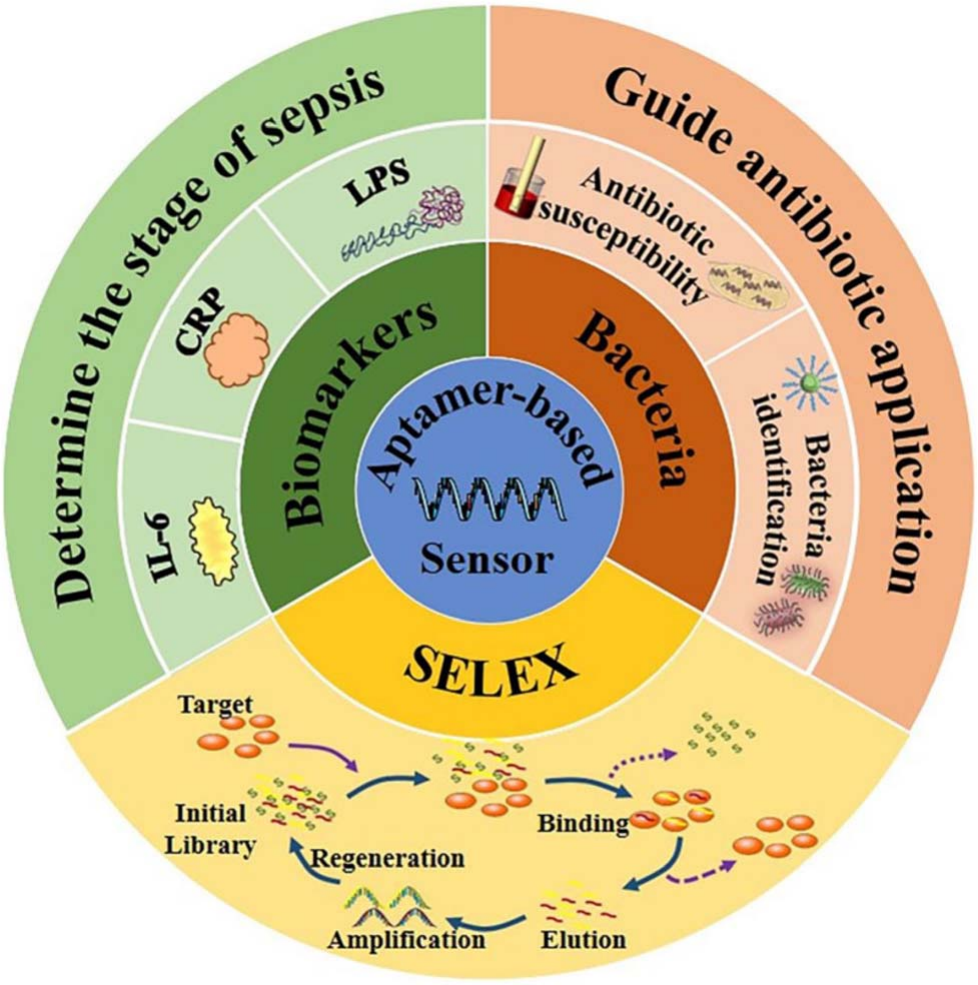
Aptamer-based biosensing strategies for sepsis diagnosis and bacterial detection. The schematic illustrates a multifunctional platform integrating aptamer-based sensors for sepsis-related biomarker detection (CRP, IL-6, and LPS), enabling stage determination and guiding antibiotic application. The SELEX (systematic evolution of ligands by exponential enrichment) process generates high-affinity aptamers through iterative binding, elution, amplification, and regeneration. These aptamers are employed for both biomarker sensing and bacterial identification. Signal amplification is achieved using aptamer-functionalized gold nanoparticles (GNPs) and magnetic bead-assisted purification, enhancing detection sensitivity under laser excitation.^319^ (Reproduced with permission from Elsevier, *Biosensors and Bioelectronics*, Copyright 2021, Elsevier B.V.).

Graziani *et al.* developed aptamers with higher sensitivity and specificity, Antibac1 & Antibac2, targeting the ubiquitous bacterial peptidoglycan.^318^ Systematic evolution of ligands by exponential enrichment (SELEX) is a method for isolating RNA or DNA molecules that bind to specific targets. In 1990, Ellington and Szostak successfully screened out oligonucleotides and named the “aptamer”, which is the first time to get an aptamer from RNA library through this method. SELEX can be used to isolate aptamers that bind to a wide range of targets, including proteins, cells, viruses, microorganisms, toxins, and chemical compounds. Aptamers generated using SELEX can be used in disease diagnosis and therapeutic approaches. SELEX is an iterative process that involves creating random pools of RNA or DNA, challenging them to bind to a target, and separating the successful binders from those that failed. The successful binders are then amplified. Fig. 17 shows the aptamer-based sepsis detection.

Zeng and group used bead-based amplification in the detection of *S. aureus* using aptamer-conjugated GNPs.^319^ Xu *et al.* realized the detection for methicillin-resistant *Staphylococcus aureus* (MRSA) using a dual-functional aptamer and CRISPR-Cas12a-assisted RCAJ Microbial Methods.^320^ Fukuzumi *et al.* used AuNPs for the detection of CRPs by decreasing the intensity of photoluminescence.^321^ Hence, researchers have used fiber-optic biosensors and microfluidic chips for LPS detection on a Nafion membrane^322^ Ferreira *et al.* developed aptamer-based sensors for the recognition of Antibac1 and Antibac2, which have higher affinity for *E. coli* and *S. aureus*.^323^ These are high-efficiency binding aptamers for a wide range of bacterial sepsis agents.

## 6 Discussion

The onset of septic infection brings in a lot of complexity, causing around 11 million deaths annually during treatments, and hence, its rapid, early diagnosis is important.^2^ The morbidity associated with sepsis is highest in critical care, resulting in prolonged ICU stays, multiorgan failure, disability, cognitive impairment and death. Unlike myocardial infarction and stroke, sepsis lacks a standard procedure, qSOFA score being simple but has limited sensitivity, whereas blood culture takes 48–72 hours.^324^ Hence, the traditional methods are resource-intensive and time-consuming, delaying critical intervention. Therefore, in the current scenario, we need a fast, less resource-dependent sensor based on fishing specific biomarkers, so that the detection process can be optimized. Currently, with the advancement in various sensors like electrochemical, optical, microchannel, and lateral flow, we are targeting very specific biomarkers in blood or even pathogens that are directly separated. With the use of PCR and LAMP, even a small amount of pathogens is detectable. Now various external stimuli (like acoustic, thermal) used in lateral flow and microchannel flow for specifically trapping the biomarkers. Using machine learning methods with optical methods of detection, we can now use fluorescence data for biomarkers for fast and reliable detection. Overall, the whole process of sepsis detection has come along with the development of various sensors and has made the whole process fast and reliable.

## 7 Challenges and future scope

Significant progress has been made in the treatment of sepsis patients; nonetheless, the disease is still linked to high rates of mortality and severe long-term cognitive impairment. Extensive research is being done in this field to validate biomarkers, make it easier to diagnose sepsis, and enable an early response that can lower the chance of death. Sometimes, a hyperinflammatory response pattern is seen in sepsis, and an immunosuppressive phase characterized by the dysfunction of many organs may ensue. A panel of biomarkers or a biomarker alone may offer a novel way to detect, diagnose, or treat sepsis. One of the main challenges in detecting sepsis from the whole blood is that the sepsis trials are predominantly conducted in high-income countries;^325^ (b) rise in awareness worldwide. They are occurring on a large scale in developing or underdeveloped countries. The challenge at such places lies in the lack of training and facilities, along with delay and lack of treatment worsening the situation. Point-of-care (POC) detection many times requires pathogen detection from blood without even processing the blood, and all the components come into play and interfere with the detection, hence we need a safer and more reliable method for sample collection and ensuring the viability of biomarker(s). Table 16 presents the key challenges and their possible solutions.

**Table 16:** Key challenges in sepsis biosensing and their potential solutions

Platform	Key challenge	Potential solution
Nanomaterial-based	Reproducibility & biofouling, expensive	Standardized synthesis, surface passivation, looking for other options like hair dye
LFA	Low sensitivity, qualitative output	Nanomaterial signal amplifiers, AI-based readout
EIS	Signal drift, complex data, low sensitivity without nanomaterials	Conductive coatings, ML-assisted data correction
Optical	Scattering, bulky and expensive setup	NIR materials, integrated photonic chips
Electrochemical	Biofouling, cross-reactivity	Antifouling layers, aptameric recognition
Microchannel	Clogging, integration	Viscoelastic separation, surface coatings
ML-based	Small datasets, interpretability	Simple model of ML
Aptameric	Stability, regeneration	Modified nucleotides; photothermal desorption

Another crucial factor downplaying the sensing comprises inhibitory factors, like blood components and primers. However, in recent times, there has been work going on to avoid blood components during pathogen detection. The unavailability of various resources like sophisticated chemicals and electricity and lack of time to perform tests make POC detection very challenging. Thus, to overcome these challenges, various advanced fabrication methods like plasma, chemical and laser treatment^326,327^ are generally combined with additive manufacturing^327^ to prepare sensing surfaces. Using these methods, microfluidic channels can be fabricated which can run efficiently and quickly to navigate through the above-mentioned problems and standardize the process leading to high fidelity of process and sensors. In such cases, the use of energy in various forms like acoustics and thermal can become crucial for pathogen separation in the microchannel. After these steps, the biosensors should be integrated with a healthcare system, so that the overall effectiveness and patient health tracking can be done easily. Overall, reducing the Limit of Detection (LOD) to ensure early detection along with ensuring the false positive & false negative results. Currently with the onset of IoT (Internet of Things)-based devices, microfluidic patches, wearables, *etc.*, are becoming prominent and these methods of sepsis detection will be important for responsible and reliable detection. Recently, multi-omics-based approach for sepsis detection is becoming more relevant as more amount of work is being done in the direction in last few years, it combines involvement of genomics, transcriptomics, proteomics, and metabolomics, making the detection results more reliable and combining it with POC will make it more useful with time.

## Conflicts of interest

There are no conflicts to declare.

## Data Availability

No new data were generated or analyzed in this study. Data sharing is not applicable to this article.

## References

[cit1] Geroulanos S., Douka E. T. (2006). Historical perspective of the word “sepsis”. Intensive Care Med..

[cit2] Rudd K. E. (2020). *et al.*, Global, regional, and national sepsis incidence and mortality, 1990–2017: analysis for the Global Burden of Disease Study. Lancet.

[cit3] Faix J. D. (2013). Biomarkers of sepsis. Crit. Rev. Clin. Lab. Sci..

[cit4] Ashley B. K., Hassan U. (2021). Point-of-critical-care diagnostics for sepsis enabled by multiplexed micro and nanosensing technologies. Wiley Interdiscip. Rev.: Nanomed. Nanobiotechnol..

[cit5] Bhatt G., Mishra K., Ramanathan G., Bhattacharya S. (2019). Dielectrophoresis assisted impedance spectroscopy for detection of gold-conjugated amplified DNA samples. Sens. Actuators, B.

[cit6] Jain U. , Chauhan N. and Saxena K., Fundamentals of sensors and biosensors: An overview, in Multifaceted Bio-sensing Technology, Elsevier, 2023, pp. 31–44, 10.1016/B978-0-323-90807-8.00005-1

[cit7] Idil N. and Mattiasson B., Electrochemical biosensors for virus detection, in Detection of viruses using electrochemical biosensors, IOP Publishing, 2021, pp. 3–1, 10.1088/978-0-7503-3849-7ch3

[cit8] Rashiku B. C. , Manoharan K, Gangopadhyay S, Bhattacharya S. Automated line detection on lateral flow assays: A paradigm shift in rapid diagnostic testing., Proceedings of the IEEE Sensors Conference (SENSORS), 2023, 10.1109/SENSORS56945.2023.10325215

[cit9] Flynn C. D., Chang D. (2023). Artificial intelligence in point-of-care biosensing: Challenges and opportunities. Biosensors.

[cit10] Kadri S., Davis A., Clancy C., Payne J. D., Klejst S. (2020). Frontlines of COVID-19: Lead, learn and cure. Process Saf. Prog..

[cit11] Singer M. (2016). *et al.*, The third international consensus definitions for sepsis and septic shock (sepsis-3). JAMA.

[cit12] Thorrington D., Andrews N., Stowe J., Miller E., van Hoek A. J. (2018). Elucidating the impact of the pneumococcal conjugate vaccine programme on pneumonia, sepsis and otitis media hospital admissions in England using a composite control. BMC Med..

[cit13] Martischang R., Pires D., Masson-Roy S., Saito H., Pittet D. (2018). Promoting and sustaining a historical and global effort to prevent sepsis: The 2018 World Health Organization SAVE LIVES: Clean Your Hands campaign. Crit. Care.

[cit14] Wong H. R. (2016). *et al.*, Pediatric Sepsis Biomarker Risk Model-II: Redefining the Pediatric Sepsis Biomarker Risk Model with Septic Shock Phenotype. Crit. Care Med..

[cit15] Cavaillon J., Singer M., Skirecki T. (2020). Sepsis therapies: learning from 30 years of failure of translational research to propose new leads. EMBO Mol. Med..

[cit16] Qu K. (2022). *et al.*, Mitochondrial dysfunction in vascular endothelial cells and its role in atherosclerosis. Front. Physiol..

[cit17] Todi S. (2024). *et al.*, A multicentre prospective registry of one thousand sepsis patients admitted in Indian ICUs: (SEPSIS INDIA) study. Crit. Care.

[cit18] Rhodes A. (2017). *et al.*, Surviving Sepsis Campaign: International Guidelines for Management of Sepsis and Septic Shock: 2016. Intensive Care Med..

[cit19] Chen W. (2017). *et al.*, Mobile Platform for Multiplexed Detection and Differentiation of Disease-Specific Nucleic Acid Sequences, Using Microfluidic Loop-Mediated Isothermal Amplification and Smartphone Detection. Anal. Chem..

[cit20] Koch C. (2020). *et al.*, Comparison of qSOFA score, SOFA score, and SIRS criteria for the prediction of infection and mortality among surgical intermediate and intensive care patients. World J. Emerg. Surg..

[cit21] Kaukonen K.-M., Bailey M., Pilcher D., Cooper D. J., Bellomo R. (2015). Systemic Inflammatory Response Syndrome Criteria in Defining Severe Sepsis. N. Engl. J. Med..

[cit22] Umemura Y. (2021). *et al.*, Current spectrum of causative pathogens in sepsis: A prospective nationwide cohort study in Japan. Int. J. Infect. Dis..

[cit23] Sakr Y. (2018). *et al.*, Sepsis in intensive care unit patients: Worldwide data from the intensive care over nations audit. Open Forum Infect. Dis..

[cit24] Shappell C., Rhee C., Klompas M. (2023). Update on Sepsis Epidemiology in the Era of COVID-19. Semin. Respir. Crit. Care Med..

[cit25] Ramachandran G. (2014). Gram-positive and gram-negative bacterial toxins in sepsis: A brief review. Virulence.

[cit26] Lin G. L., McGinley J. P., Drysdale S. B., Pollard A. J. (2018). Epidemiology and Immune Pathogenesis of Viral Sepsis. Front. Immunol..

[cit27] Boussina A. , Ramesh K., Arora H., Ratadiya P. and Nemati S., ‘Differentiation of Fungal, Viral, and Bacterial Sepsis using Multimodal Deep Learning’, medRxiv, 2023, preprint, 10.1101/2023.04.10.23288378

[cit28] Bergin S. P. (2015). *et al.*, Neonatal Escherichia coli Bloodstream Infections: Clinical Outcomes and Impact of Initial Antibiotic Therapy. Pediatr. Infect. Dis. J..

[cit29] Chen Y. (2020). *et al.*, Preterm infants harbour diverse klebsiella populations, including atypical species that encode and produce an array of antimicrobial resistance-and virulence-associated factors. Microb. Genom..

[cit30] Hernandez-Alonso E. (2022). *et al.*, Contaminated Incubators: Source of a Multispecies Enterobacter Outbreak of Neonatal Sepsis. Microbiol. Spectr..

[cit31] Linz M. S., Mattappallil A., Finkel D., Parker D. (2023). Clinical Impact of Staphylococcus aureus Skin and Soft Tissue Infections. Antibiotics.

[cit32] Harris K., Proctor L. K., Shinar S., Philippopoulos E., Yudin M. H., Murphy K. E. (2023). Outcomes and management of pregnancy and puerperal group A streptococcal infections: A systematic review. Acta Obstet. Gynecol. Scand..

[cit33] Jarczak D., Kluge S., Nierhaus A. (2021). Sepsis—Pathophysiology and Therapeutic Concepts. Front. Med..

[cit34] Soni M., Handa M., Singh K. K., Shukla R. (2022). Recent nanoengineered diagnostic and therapeutic advancements in management of Sepsis. J. Contr. Release.

[cit35] Schouten M., Wiersinga W. J., Levi M., van der Poll T. (2008). Inflammation, endothelium, and coagulation in sepsis. J. Leukoc. Biol..

[cit36] Arcel M. and Evi L., ‘Disseminated Intravascular Coagulation’, 1999

[cit37] Dixon B. , ‘The Role of Microvascular Thrombosis in Sepsis’, 2004 10.1177/0310057X0403200502 15535483

[cit38] Boomer J. S. (2011). *et al.*, Immunosuppression in patients who die of sepsis and multiple organ failure. JAMA.

[cit39] Vivier E., Malissen B. (2005). Innate and adaptive immunity: Specificities and signaling hierarchies revisited. Nat. Immunol..

[cit40] Soni M., Handa M., Singh K. K., Shukla R. (2022). Recent nanoengineered diagnostic and therapeutic advancements in management of Sepsis. J. Contr. Release.

[cit41] Srivastava A., Mannam P. (2015). Warburg revisited: Lessons for innate immunity and sepsis. Front. Physiol..

[cit42] Kumar S., Tripathy S., Singh O. K., Singh S. G. (2021). Cerium oxide nanofiber based electroanalytical sensor for TNF-α detection: Improved interfacial stability with Nafion. Bioelectrochemistry.

[cit43] Tsounidi D. (2021). *et al.*, Development of a point-of-care system based on white light reflectance spectroscopy: Application in crp determination. Biosensors.

[cit44] Karakike E. (2019). *et al.*, Late Peaks of HMGB1 and Sepsis Outcome: Evidence for Synergy with Chronic Inflammatory Disorders. Shock.

[cit45] Thongkhao P., Numnuam A., Khongkow P., Sangkhathat S., Phairatana T. (2024). Disposable Polyaniline/m-Phenylenediamine-Based Electrochemical Lactate Biosensor for Early Sepsis Diagnosis. Polymers.

[cit46] Worthington T. , Lambert P. A. and Traube A., ‘A rapid ELISA for the diagnosis of intravascular catheter related sepsis caused by coagulase negative staphylococci’, 2002, available: https://www.jclinpath.com 10.1136/jcp.55.1.41 PMC1769565 11825923

[cit47] Mahboob S., Iqbal M., Rahman M. (2023). Development of ELISA-based diagnostic methods for the detection of haemorrhagic septicaemia in animals. J. Microbiol. Methods.

[cit48] Liao T., Yuan F., Yu H., Li Z. (2016). An ultrasensitive ELISA method for the detection of procalcitonin based on magnetic beads and enzyme-antibody labeled gold nanoparticles. Anal. Methods.

[cit49] Verma M. S. (2018). *et al.*, Sliding-strip microfluidic device enables ELISA on paper. Biosens. Bioelectron..

[cit50] Zhao Z. (2024). *et al.*, A retrospective study of the detection of sepsis pathogens comparing blood culture and culture-independent digital PCR. Heliyon.

[cit51] Tsantes A. G. (2023). *et al.*, Sepsis-induced coagulopathy: An update on pathophysiology, biomarkers, and current guidelines. Life.

[cit52] Califf R. M. (2018). Biomarker definitions and their applications. Exp. Biol. Med..

[cit53] Mayr F. B., Yende S., Angus D. C. (2014). Epidemiology of severe sepsis. Virulence.

[cit54] Lee S. (2022). *et al.*, Diagnostic and prognostic value of presepsin and procalcitonin in non-infectious organ failure, sepsis, and septic shock: a prospective observational study according to the Sepsis-3 definitions. BMC Infect. Dis..

[cit55] Hung S. K., Lan H. M., Han S. T., Wu C. C., Chen K. F. (2020). Current evidence and limitations of biomarkers for detecting sepsis and systemic infection. Biomedicines.

[cit56] Önal U., Valenzuela-Sánchez F., Vandana K. E., Rello J. (2018). Mid-regional pro-adrenomedullin (MR-proADM) as a biomarker for sepsis and septic shock: A narrative review. Healthcare.

[cit57] Nakajima A. (2014). *et al.*, Clinical utility of procalcitonin as a marker of sepsis: A potential predictor of causative pathogens. Intern. Med..

[cit58] Mohammed M. A. (2023). Fighting cytokine storm and immunomodulatory deficiency: Using natural products therapy up to now. Front. Pharmacol..

[cit59] Yeh C. F., Wu C. C., Liu S. H., Chen K. F. (2019). Comparison of the accuracy of neutrophil CD64, procalcitonin, and C-reactive protein for sepsis identification: a systematic review and meta-analysis. Ann. Intensive Care.

[cit60] Yende S. (2019). *et al.*, Long-term Host Immune Response Trajectories among Hospitalized Patients with Sepsis. JAMA Netw. Open.

[cit61] Zhang Y., Feng Q., Zhou S., Chen H., Bakir M. (2020). Downregulation of serum survivin correlates with increased inflammation, enhanced disease severity and worse prognosis in sepsis patients. Medicine.

[cit62] Tian R. (2019). *et al.*, Plasma PTX3, MCP1 and Ang2 are early biomarkers to evaluate the severity of sepsis and septic shock. Scand. J. Immunol..

[cit63] Hamed S. (2017). *et al.*, Diagnostic value of Pentraxin-3 in patients with sepsis and septic shock in accordance with latest sepsis-3 definitions. BMC Infect. Dis..

[cit64] Hoppensteadt D., Tsuruta K., Hirman J., Kaul I., Osawa Y., Fareed J. (2015). Dysregulation of inflammatory and hemostatic markers in sepsis and suspected disseminated intravascular coagulation. Clin. Appl. Thromb. Hemost..

[cit65] Yende S. (2019). *et al.*, Long-term Host Immune Response Trajectories among Hospitalized Patients with Sepsis. JAMA Netw. Open.

[cit66] Matsumoto H. (2018). *et al.*, The clinical importance of a cytokine network in the acute phase of sepsis. Sci. Rep..

[cit67] Hesse R. (2016). *et al.*, Decreased IL-8 levels in CSF and serum of AD patients and negative correlation of MMSE and IL-1β. BMC Neurol..

[cit68] Larsson A. (2020). *et al.*, Calprotectin is superior to procalcitonin as a sepsis marker and predictor of 30-day mortality in intensive care patients. Scand. J. Clin. Lab. Invest..

[cit69] Karlsson S., Pettilä V., Tenhunen J., Laru-Sompa R., Hynninen M., Ruokonen E. (2008). HMGB1 as a predictor of organ dysfunction and outcome in patients with severe sepsis. Intensive Care Med..

[cit70] Fang Y., Li C., Shao R., Yu H., Zhang Q. (2018). The role of biomarkers of endothelial activation in predicting morbidity and mortality in patients with severe sepsis and septic shock in intensive care: A prospective observational study. Thromb. Res..

[cit71] Barichello T., Generoso J. S., Singer M., Dal-Pizzol F. (2022). Biomarkers for sepsis: More than just fever and leukocytosis—a narrative review. Crit. Care.

[cit72] Erikson K. (2020). *et al.*, Brain tight junction protein expression in sepsis in an autopsy series. Crit. Care.

[cit73] Wu L. (2020). *et al.*, The dynamic change of serum S100B levels from day 1 to day 3 is more associated with sepsis-associated encephalopathy. Sci. Rep..

[cit74] Yao B., Zhang L. N., Ai Y. H., Liu Z. Y., Huang L. (2014). Serum S100β is a better biomarker than neuron-specific enolase for sepsis-associated encephalopathy and determining its prognosis: A prospective and observational study. Neurochem. Res..

[cit75] Skibsted S. (2013). *et al.*, Biomarkers of endothelial cell activation in early sepsis. Shock.

[cit76] Crenn P. (2014). *et al.*, Plasma l-citrulline concentrations and its relationship with inflammation at the onset of septic shock: A pilot study. J. Crit. Care.

[cit77] Ware L. B. (2013). *et al.*, Low plasma citrulline levels are associated with acute respiratory distress syndrome in patients with severe sepsis. Crit. Care.

[cit78] Li J., Ren Y., Gao C., Zhang K., Zheng F., Kang J. (2021). Evaluation of Fecal Calprotectin, D-Lactic Acid and Bedside Gastrointestinal Ultrasound Image Data for the Prediction of Acute Gastrointestinal Injury in Sepsis Patients. Front. Med. Technol..

[cit79] Zhao D., Li S., Cui J., Wang L., Ma X., Li Y. (2020). Plasma miR-125a and miR-125b in sepsis: Correlation with disease risk, inflammation, severity, and prognosis. J. Clin. Lab. Anal..

[cit80] Zhu X. (2020). MiR-125b but not miR-125a is
upregulated and exhibits a trend to correlate with enhanced disease severity, inflammation, and increased mortality in sepsis patients. J. Clin. Lab. Anal..

[cit81] Gui F., Peng H., Liu Y. (2019). Elevated circulating lnc-ANRIL/miR-125a axis level predicts higher risk, more severe disease condition, and worse prognosis of sepsis. J. Clin. Lab. Anal..

[cit82] Chen K., Shi X., Jin Y., Wang F., Shen Q., Xu W. (2019). High lncRNA MEG3 expression is associated with high mortality rates in patients with sepsis and increased lipopolysaccharide-induced renal epithelial cell and cardiomyocyte apoptosis. Exp. Ther. Med..

[cit83] Yin W. P., Li J. B., Zheng X. F., An L., Shao H., Li C. S. (2020). Effect of neutrophil CD64 for diagnosing sepsis in emergency department. World J. Emerg. Med..

[cit84] Westhoff D. (2019). *et al.*, Systemic infection and microglia activation: A prospective postmortem study in sepsis patients. Immun. Ageing.

[cit85] Ehler J. (2019). *et al.*, The prognostic value of neurofilament levels in patients with sepsis-associated encephalopathy – A prospective, pilot observational study. PLoS One.

[cit86] Lu B. (2018). *et al.*, The utility of presepsin in diagnosis and risk stratification for the emergency patients with sepsis. Am. J. Emerg. Med..

[cit87] Aksaray S. (2016). Diagnostic value of sTREM-1 and procalcitonin levels in the early diagnosis of sepsis. North. Clin. Istanb..

[cit88] Şen S. (2021). *et al.*, Surface TREM-1 as a Prognostic Biomarker in Pediatric Sepsis. Indian J. Pediatr..

[cit89] Casagranda I. (2015). *et al.*, Usefulness of suPAR in the risk stratification of patients with sepsis admitted to the emergency department. Intern. Emerg. Med..

[cit90] Spoto S. (2018). *et al.*, Procalcitonin and MR-Proadrenomedullin Combination with SOFA and qSOFA Scores for Sepsis Diagnosis and Prognosis: A Diagnostic Algorithm. Shock.

[cit91] Yang Y. (2020). *et al.*, Development of a nomogram to predict 30-day mortality of patients with sepsis-associated encephalopathy: A retrospective cohort study. J. Intensiv. Care.

[cit92] Maruchi Y. (2018). *et al.*, Plasma myeloperoxidase-conjugated DNA level predicts outcomes and organ dysfunction in patients with septic shock. Crit. Care.

[cit93] Bonaventura A. (2020). *et al.*, The role of resistin and myeloperoxidase in severe sepsis and septic shock: Results from the ALBIOS trial. Eur. J. Clin. Invest..

[cit94] Karampela I. (2019). *et al.*, Circulating eNampt and resistin as a proinflammatory duet predicting independently mortality in critically ill patients with sepsis: A prospective observational study. Cytokine.

[cit95] Liu M. (2017). *et al.*, Serum sPD-L1, Upregulated in Sepsis, May Reflect Disease Severity and Clinical Outcomes in Septic Patients. Scand. J. Immunol..

[cit96] Feng Q. P. (2019). *et al.*, Association Between Low-Density Lipoprotein Cholesterol Levels and Risk for Sepsis Among Patients Admitted to the Hospital With Infection. JAMA Netw. Open.

[cit97] Chien J. Y., Jerng J. S., Yu C. J., Yang P. C. (2005). Low serum level of high-density lipoprotein cholesterol is a poor prognostic factor for severe sepsis. Crit. Care Med..

[cit98] Jongjinakool S. , Palasak K., Bousod N. and Teepoo S., ‘Gold nanoparticles-based colorimetric sensor for cysteine detection’, in Energy Procedia, Elsevier Ltd, 2014, pp. 10–18, 10.1016/j.egypro.2014.07.126

[cit99] Teengam P., Siangproh W., Tuantranont A., Vilaivan T., Chailapakul O., Henry C. S. (2017). Multiplex Paper-Based Colorimetric DNA Sensor Using Pyrrolidinyl Peptide Nucleic Acid-Induced AgNPs Aggregation for Detecting MERS-CoV, MTB, and HPV Oligonucleotides. Anal. Chem..

[cit100] Byun J. Y., Shin Y. B., Kim D. M., Kim M. G. (2013). A colorimetric homogeneous immunoassay system for the C-reactive protein. Analyst.

[cit101] Kim K. M., Nguyen P. T., Kim J., Song S. H., Park J. W., Il Kim M. (2024). Chemiluminescence Immunoassay for Sensitive Detection of C-reactive Protein Using Graphene Oxide–Gold Nanoparticle–Luminol Hybrids as Enhanced Luminogenic Molecules. Chemosensors.

[cit102] Wang M. (2024). *et al.*, Advancements in magnetic nanoparticle-based biosensors for point-of-care testing. Front. Bioeng. Biotechnol..

[cit103] Herrmann I. K. (2013). *et al.*, Endotoxin removal by magnetic separation-based blood purification. Adv. Healthc. Mater..

[cit104] Reddy B. (2018). *et al.*, Point-of-care sensors for the management of sepsis. Nat. Biomed. Eng..

[cit105] Ershad M., Mostafa A., Dela Cruz M., Vearrier D. (2019). Neonatal Sepsis. Curr. Emerg. Hosp. Med. Rep..

[cit106] Balayan S., Chauhan N., Chandra R., Jain U. (2022). Molecular imprinting based electrochemical biosensor for identification of serum amyloid A (SAA), a neonatal sepsis biomarker. Int. J. Biol. Macromol..

[cit107] Gopal N., Chauhan N., Jain U., Dass S. K., Sharma H. S., Chandra R. (2023). Advancement in biomarker-based effective diagnosis of neonatal sepsis. RNA Biology.

[cit108] Kumar D., Prasad B. B. (2012). Multiwalled carbon nanotubes embedded molecularly imprinted polymer-modified screen printed carbon electrode for the quantitative analysis of C-reactive protein. Sens. Actuators, B.

[cit109] Justino C. I. L., Freitas A. C., Amaral J. P., Rocha-Santos T. A. P., Cardoso S., Duarte A. C. (2013). Disposable immunosensors for C-reactive protein based on carbon nanotubes field effect transistors. Talanta.

[cit110] Bryan T., Luo X., Bueno P. R., Davis J. J. (2013). An optimised electrochemical biosensor for the label-free detection of C-reactive protein in blood. Biosens. Bioelectron..

[cit111] Thangamuthu M., Santschi C., Martin O. J. F. (2018). Label-free electrochemical immunoassay for C-reactive protein. Biosensors.

[cit112] Pandiaraj M. (2013). *et al.*, Nanomaterial-based electrochemical biosensors for cytochrome c using cytochrome c reductase. Bioelectrochemistry.

[cit113] Qureshi A., Gurbuz Y., Kallempudi S., Niazi J. H. (2010). Label-free RNA aptamer-based capacitive biosensor for the detection of C-reactive protein. Phys. Chem. Chem. Phys..

[cit114] Liu X. (2023). *et al.*, Selection of a Novel DNA Aptamer Specific for 5-Hydroxymethylfurfural Using Capture-SELEX. Biosensors.

[cit115] Qi H., Zhang C. (2020). Electrogenerated chemiluminescence biosensing. Anal. Chem..

[cit116] Jimenez V. O. (2022). *et al.*, Magnetoimpedance biosensors and real-time healthcare monitors: Progress, opportunities, and challenges. Biosensors.

[cit117] Martens D. (2018). *et al.*, A low-cost integrated biosensing platform based on SiN nanophotonics for biomarker detection in urine. Anal. Methods.

[cit118] Ma Y. (2020). *et al.*, Electrochemical detection of C-reactive protein using functionalized iridium nanoparticles/graphene oxide as a tag. RSC Adv..

[cit119] Liu C. (2016). *et al.*, Plasmonic ZnO nanorods/Au substrates for protein microarrays with high sensitivity and broad dynamic range. Sens. Actuators, B.

[cit120] Kim H. M., Uh M., Jeong D. H., Lee H. Y., Park J. H., Lee S. K. (2019). Localized surface plasmon resonance biosensor using nanopatterned gold particles on the surface of an optical fiber. Sens. Actuators, B.

[cit121] Sridevi S., Vasu K. S., Asokan S., Sood A. K. (2015). Sensitive detection of C-reactive protein using optical fiber Bragg gratings. Biosens. Bioelectron..

[cit122] Sai V. V. R., Kundu T., Deshmukh C., Titus S., Kumar P., Mukherji S. (2010). Label-free fiber optic biosensor based on evanescent wave absorbance at 280 nm. Sens. Actuators, B.

[cit123] Bing X., Wang G. (2017). Label free C-reactive protein detection based on an electrochemical sensor for clinical application. Int. J. Electrochem. Sci..

[cit124] Shi S. (2015). *et al.*, A polydopamine-modified optical fiber SPR biosensor using electroless-plated gold films for immunoassays. Biosens. Bioelectron..

[cit125] Sager R., Kutz A., Mueller B., Schuetz P. (2017). Procalcitonin-guided diagnosis and antibiotic stewardship revisited. BMC Med..

[cit126] Lim J. M., Ryu M. Y., Kim J. H., Cho C. H., Park T. J., Park J. P. (2017). An electrochemical biosensor for detection of the sepsis-related biomarker procalcitonin. RSC Adv..

[cit127] Tanak A. S., Jagannath B., Tamrakar Y., Muthukumar S., Prasad S. (2019). Non-faradaic electrochemical impedimetric profiling of procalcitonin and C-reactive protein as a dual marker biosensor for early sepsis detection. Anal. Chim. Acta: X.

[cit128] Fang Y. S., Wang H. Y., Wang L. S., Wang J. F. (2014). Electrochemical immunoassay for procalcitonin antigen detection based on signal amplification strategy of multiple nanocomposites. Biosens. Bioelectron..

[cit129] Baldini F. (2009). *et al.*, A new procalcitonin optical immunosensor for POCT applications. Anal. Bioanal. Chem..

[cit130] Szymanska B., Lukaszewski Z., Oldak L., Zelazowska-Rutkowska B., Hermanowicz-Szamatowicz K., Gorodkiewicz E. (2022). Two Biosensors for the Determination of Interleukin-6 in Blood Plasma by Array SPRi. Biosensors.

[cit131] Liu A. and Wang X., ‘Amperometric Immunosensor of Procalcitonin Based on Amplification Strategy of Ferrocene-Modified Gold nanoparticles’, 2015, available: https://www.electrochemsci.org

[cit132] Chen P. (2015). *et al.*, Multiplex serum cytokine immunoassay using nanoplasmonic biosensor microarrays. ACS Nano.

[cit133] Arnon S., Litmanovitz I., Regev R. H., Bauer S., Shainkin-Kestenbaum R., Dolfin T. (2007). Serum amyloid A protein in the early detection of late-onset bacterial sepsis in preterm infants. J. Perinatol..

[cit134] Behrendt D., Dembinski J., Heep A., Bartmann P. (2004). Lipopolysaccharide binding protein in preterm infants. Arch. Dis. Child. Fetal Neonatal Ed..

[cit135] Pui T. S., Kongsuphol P., Arya S. K., Bansal T. (2013). Detection of tumor necrosis factor (TNF-α) in cell culture medium with label free electrochemical impedance spectroscopy. Sens. Actuators, B.

[cit136] Arkusz K., Paradowska E. (2020). Impedimetric detection of femtomolar levels of interleukin6, interleukin 8, and tumor necrosis factor alpha based on thermally modified nanotubular titanium dioxide arrays. Nanomaterials.

[cit137] Rashidova G., Tilegen M., Pham T. T., Bekmurzayeva A., Tosi D. (2024). Functionalized optical fiber ball-shaped biosensor for label-free, low-limit detection of IL-8 protein. Biomed. Opt. Express.

[cit138] Chen H. (2021). *et al.*, Pentraxin-3 is a strong biomarker of sepsis severity identification and predictor of 90-day mortality in intensive care units via sepsis 3.0 definitions. Diagnostics.

[cit139] Sharma R. (2016). *et al.*, Label-free electrochemical impedance biosensor to detect human interleukin-8 in serum with sub-pg/ml sensitivity. Biosens. Bioelectron..

[cit140] Huu Tien C. , et al., ‘Detection of Interleukin-8 mRNA Using a Gold Nanowire-Based Biosensor with a Stem-Loop Probe’, 1981

[cit141] Centi S., Tombelli S., Puntoni M., Domenici C., Franek M., Palchetti I. (2015). Detection of biomarkers for inflammatory diseases by an electrochemical immunoassay: The case of neopterin. Talanta.

[cit142] Santelli G. , et al., ‘Urinary Neopterin and Immunological Features in Patients with Kaposi’s Sarcoma’ 10.1016/0277-5379(88)90327-6 3263274

[cit143] Koryakina A., Frey E., Bruegger P. (2014). Cryopreservation of human monocytes for pharmacopeial monocyte activation test. J. Immunol. Methods.

[cit144] Alqahtani A. (2026). Clinical evaluation of plasma neopterin as a biomarker of immune activation using a fluorescence-based o-phthaldehyde derivatization method. Sci. Rep..

[cit145] El-Madbouly A. A., El Sehemawy A. A., Eldesoky N. A., Abd Elgalil H. M., Ahmed A. M. (2019). Utility of presepsin, soluble triggering receptor expressed on myeloid cells-1, and neutrophil CD64 for early detection of neonatal sepsis. Infect. Drug Resist..

[cit146] Daramola O. A., Heyderman R. S., Klein N. J., Shennan G. I., Levin M. (1997). Detection of fibronectin expression by human endothelial cells using an enzyme-linked immunosorbent assay (ELISA): enzymatic degradation by activated plasminogen. J. Immunol. Methods.

[cit147] Tatiya S., Pandey M., Bhattacharya S. (2020). Nanoparticles containing boron and its compounds—synthesis and applications: A review. J. Micromanufacturing.

[cit148] Tatiya S., Pandey M., Gupta S., Bhattacharya S. (2023). Photocatalytic Performance of Boron-Doped TiO2 for Treatment of Rhodamine-B Dye and Industrial Wastewater Under Ultraviolet Irradiation. Environ. Eng. Sci..

[cit149] Manoharan K. , Pandey M. and Bhattacharya S., ‘Life cycle assessment of nanoscale polymer-based coatings’, in Polymer-Based Nanoscale Materials for Surface Coatings, Elsevier, 2023, pp. 613–631, 10.1016/B978-0-32-390778-1.00033-5

[cit150] Pandey M. , Sundriyal P., Tatiya S. and Bhattacharya S., ‘Polymer-Based Electrolytes for Solid-State Batteries: Current Status and Future Challenges in Emerging Applications’, in Trends in Applications of Polymers and Polymer Composites, AIP Publishing, 2022, pp. 1–22, 10.1063/9780735424555_005

[cit151] Dubey A. (2018). *et al.*, An eco-friendly, low-power charge storage device from bio-tolerable nano cerium oxide electrodes for bioelectrical and biomedical applications. Biomed. Phys. Eng. Express.

[cit152] António M., Nogueira J., Vitorino R., Daniel-da-Silva A. L. (2018). Functionalized gold nanoparticles for the detection of C-reactive protein. Nanomaterials.

[cit153] Russell S. M., Alba-Patiño A., Borges M., de la Rica R. (2019). Multifunctional motion-to-color janus transducers for the rapid detection of sepsis biomarkers in whole blood. Biosens. Bioelectron..

[cit154] Fang Y., Hu Q., Yu X., Wang L. (2018). Ultrasensitive electrochemical immunosensor for procalcitonin with signal enhancement based on zinc nanoparticles functionalized ordered mesoporous carbon-silica nanocomposites. Sens. Actuators, B.

[cit155] Bonini A. (2022). *et al.*, Emerging biosensing technologies towards early sepsis diagnosis and management. Biosensors.

[cit156] Liu F. (2014). *et al.*, Procalcitonin sensitive detection based on graphene-gold nanocomposite film sensor platform and single-walled carbon nanohorns/hollow Pt chains complex as signal tags. Biosens. Bioelectron..

[cit157] Liu A. and Wang X., ‘Amperometric Immunosensor of Procalcitonin Based on Amplification Strategy of Ferrocene-Modified Gold nanoparticles’, 2015, available: https://www.electrochemsci.org

[cit158] Liu P. (2019). *et al.*, An ultrasensitive electrochemical immunosensor for procalcitonin detection based on the gold nanoparticles-enhanced tyramide signal amplification strategy. Biosens. Bioelectron..

[cit159] Li Y. (2020). *et al.*, A dual-mode PCT electrochemical immunosensor with CuCo2S4 bimetallic sulfides as enhancer. Biosens. Bioelectron..

[cit160] Molinero-Fernández Á., Moreno-Guzmán M., Arruza L., López M. Á., Escarpa A. (2019). Toward Early Diagnosis of Late-Onset Sepsis in Preterm Neonates: Dual Magnetoimmunosensor for Simultaneous Procalcitonin and C-Reactive Protein Determination in Diagnosed Clinical Samples. ACS Sens..

[cit161] Xu P. (2021). *et al.*, Electrochemiluminescence immunosensor based on ferrocene functionalized ZIF-8 quenching the electrochemiluminescence of Ru(bpy)32+-doped silica nanoparticles embodied N-butyl diethanolamine. Sens. Actuators, B.

[cit162] Fang J. (2021). *et al.*, Dual-quenching electrochemiluminescence system based on novel acceptor CoOOH@Au NPs for early detection of procalcitonin. Sens. Actuators, B.

[cit163] Jing W. (2019). *et al.*, Time-Resolved Digital Immunoassay for Rapid and Sensitive Quantitation of Procalcitonin with Plasmonic Imaging. ACS Nano.

[cit164] Li P. (2018). *et al.*, Establishment of a novel homogeneous nanoparticle-based assay for sensitive procalcitonin detection of ultra low-volume serum samples. Int. J. Nanomed..

[cit165] Nie R., Huang J., Xu X., Yang L. (2020). A portable pencil-like immunosensor for point-of-care testing of inflammatory biomarkers. Anal. Bioanal. Chem..

[cit166] Chen Y. (2017). *et al.*, Double-Enzymes-Mediated Bioluminescent Sensor for Quantitative and Ultrasensitive Point-of-Care Testing. Anal. Chem..

[cit167] Jing W. (2019). *et al.*, Time-Resolved Digital Immunoassay for Rapid and Sensitive Quantitation of Procalcitonin with Plasmonic Imaging. ACS Nano.

[cit168] Ai Y., Sanders C. K., Marrone B. L. (2013). Separation of escherichia coli bacteria from peripheral blood mononuclear cells using standing surface acoustic waves. Anal. Chem..

[cit169] Ohlsson P. (2016). *et al.*, Integrated Acoustic Separation, Enrichment, and Microchip Polymerase Chain Reaction Detection of Bacteria from Blood for Rapid Sepsis Diagnostics. Anal. Chem..

[cit170] Dow P., Kotz K., Gruszka S., Holder J., Fiering J. (2018). Acoustic separation in plastic microfluidics for rapid detection of bacteria in blood using engineered bacteriophage. Lab Chip.

[cit171] Pethig R. (2010). Dielectrophoresis: Status of the theory, technology, and applications. Biomicrofluidics.

[cit172] Kuczenski R. S., Chang H. C., Revzin A. (2011). Dielectrophoretic microfluidic device for the continuous sorting of Escherichia coli from blood cells. Biomicrofluidics.

[cit173] D'Amico L., Ajami N. J., Adachi J. A., Gascoyne P.
R. C., Petrosino J. F. (2017). Isolation and concentration of bacteria from blood using microfluidic membraneless dialysis and dielectrophoresis. Lab Chip.

[cit174] Wang S. Q. (2012). *et al.*, Portable microfluidic chip for detection of Escherichia coli in produce and blood. Int. J. Nanomed..

[cit175] Lee J. J. (2014). *et al.*, Synthetic ligand-coated magnetic nanoparticles for microfluidic bacterial separation from blood. Nano Lett..

[cit176] Yung C. W., Fiering J., Mueller A. J., Ingber D. E. (2009). Micromagnetic-microfluidic blood cleansing device. Lab Chip.

[cit177] Wu Z., Willing B., Bjerketorp J., Jansson J. K., Hjort K. (2009). Soft inertial microfluidics for high throughput separation of bacteria from human blood cells. Lab Chip.

[cit178] Martel J. M., Toner M. (2014). Inertial focusing in microfluidics. Annu. Rev. Biomed. Eng..

[cit179] Monneret G., Venet F. (2016). Sepsis-induced immune alterations monitoring by flow cytometry as a promising tool for individualized therapy. Cytometry, Part B.

[cit180] Hou H. W., Bhattacharyya R. P., Hung D. T., Han J. (2015). Direct detection and drug-resistance profiling of bacteremias using inertial microfluidics. Lab Chip.

[cit181] The 15th International Conference on Solid-State Sensors, Actuators & Microsystems : Transducers 2009, Denver, Colorado, U.S.A., June 21-25, 2009, Sheraton Denver Hotel, IEEE Electron Devices Society, 2009

[cit182] Christner M., Rohde H., Wolters M., Sobottka I., Wegscheider K., Aepfelbacher M. (2010). Rapid identification of bacteria from positive blood culture bottles by use of matrix-assisted laser desorption-ionization time of flight mass spectrometry fingerprinting. J. Clin. Microbiol..

[cit183] Vashist S. K., Schneider E. M., Barth E., Luong J. H. T. (2016). Surface plasmon resonance-based immunoassay for procalcitonin. Anal. Chim. Acta.

[cit184] Boonkaew S., Jang I., Noviana E., Siangproh W., Chailapakul O., Henry C. S. (2021). Electrochemical paper-based analytical device for multiplexed, point-of-care detection of cardiovascular disease biomarkers. Sens. Actuators, B.

[cit185] Gao J. (2017). *et al.*, A Multiplex Electrochemical Biosensor for Bloodstream Infection Diagnosis. SLAS Technol..

[cit186] Sequeira-Antunes B., Ferreira H. A. (2023). Nucleic acid aptamer-based biosensors: A review. Biomedicines.

[cit187] Yang T., Wang S., Jin H., Bao W., Huang S., Wang J. (2013). An electrochemical impedance sensor for the label-free ultrasensitive detection of interleukin-6 antigen. Sens. Actuators, B.

[cit188] Ehzari H., Samimi M., Safari M., Gholivand M. B. (2020). Label-free electrochemical immunosensor for sensitive HER2 biomarker detection using the core-shell magnetic metal-organic frameworks. J. Electroanal. Chem..

[cit189] Fan G. C., Ren X. L., Zhu C., Zhang J. R., Zhu J. J. (2014). A new signal amplification strategy of photoelectrochemical immunoassay for highly sensitive interleukin-6 detection based on TiO2/CdS/CdSe dual co-sensitized structure. Biosens. Bioelectron..

[cit190] Hu J. (2016). *et al.*, Sensitive and Quantitative Detection of C-Reaction Protein Based on Immunofluorescent Nanospheres Coupled with Lateral Flow Test Strip. Anal. Chem..

[cit191] Li H., Sun Y., Elseviers J., Muyldermans S., Liu S., Wan Y. (2014). A nanobody-based electrochemiluminescent immunosensor for sensitive detection of human procalcitonin. Analyst.

[cit192] Yuan L., Hua X., Wu Y., Pan X., Liu S. (2011). Polymer-functionalized silica nanosphere labels for ultrasensitive detection of tumor necrosis factor-alpha. Anal. Chem..

[cit193] Li H. (2021). *et al.*, Quench-Release-Based Fluorescent Immunosensor for the Rapid Detection of Tumor Necrosis Factor α. ACS Omega.

[cit194] Qian S. (2023). *et al.*, Phosphorylcholine-Functionalized PEDOT-Gated Organic Electrochemical Transistor Devices for Ultra-Specific and Sensitive C-Reactive Protein Detection. Polymers.

[cit195] Macchia E. (2019). *et al.*, Selective single-molecule analytical detection of C-reactive protein in saliva with an organic transistor. Anal. Bioanal. Chem..

[cit196] Russell S. M., Alba-Patiño A., Borges M., de la Rica R. (2019). Multifunctional motion-to-color janus transducers for the rapid detection of sepsis biomarkers in whole blood. Biosens. Bioelectron..

[cit197] Seshadri P. (2018). *et al.*, Low-picomolar, label-free procalcitonin analytical detection with an electrolyte-gated organic field-effect transistor based electronic immunosensor. Biosens. Bioelectron..

[cit198] He L. (2017). *et al.*, Label-free femtomolar cancer biomarker detection in human serum using graphene-coated surface plasmon resonance chips. Biosens. Bioelectron..

[cit199] Henne W. A., Doorneweerd D. D., Lee J., Low P. S., Savran C. (2006). Detection of folate binding protein with enhanced sensitivity using a functionalized quartz crystal microbalance sensor. Anal. Chem..

[cit200] Esteban-Fernández De Ávila B. (2013). *et al.*, Ultrasensitive amperometric magnetoimmunosensor for human C-reactive protein quantification in serum. Sens. Actuators, B.

[cit201] Pandey M. , Shahare K., Srivastava M. and Bhattacharya S., ‘Paper-Based Devices for Wearable Diagnostic Applications’, 2019, pp. 193–208, 10.1007/978-981-15-0489-1_12

[cit202] Mie G. (1908). Beiträge zur Optik trüber Medien, speziell kolloidaler Metallösungen. Ann. Phys..

[cit203] Fang Y. S., Wang H. Y., Wang L. S., Wang J. F. (2014). Electrochemical immunoassay for procalcitonin antigen detection based on signal amplification strategy of multiple nanocomposites. Biosens. Bioelectron..

[cit204] Pandey M. , Srivastava M., Shahare K. and Bhattacharya S., Paper microfluidic-based devices for infectious disease diagnostics, in Paper-Based Microfluidics, Springer, 2019, pp. 209–225, 10.1007/978-981-15-0489-1_13

[cit205] Chauhan P. S. , Pandey M. and Bhattacharya S., ‘Paper Based Sensors for Environmental Monitoring’, 2019, pp. 165–181, 10.1007/978-981-15-0489-1_10

[cit206] Prakashan D., Kolhe P., Gandhi S. (2024). Design and fabrication of a competitive lateral flow assay using gold nanoparticle as capture probe for the rapid and on-site detection of penicillin antibiotic in food samples. Food Chem..

[cit207] Bradley Z. (2023). *et al.*, Effect of Selenium Nanoparticle Size on IL-6 Detection Sensitivity in a Lateral Flow Device. ACS Omega.

[cit208] Ji T. (2020). *et al.*, Background-Free Chromatographic Detection of Sepsis Biomarker in Clinical Human Serum through Near-Infrared to Near-Infrared Upconversion Immunolabeling. ACS Nano.

[cit209] Alba-Patiño A., Russell S. M., Borges M., Pazos-Pérez N., Álvarez-Puebla R. A., De La Rica R. (2020). Nanoparticle-based mobile biosensors for the rapid detection of sepsis biomarkers in whole blood. Nanoscale Adv..

[cit210] Taratummarat S. (2018). *et al.*, Gold nanoparticles attenuates bacterial sepsis in cecal ligation and puncture mouse model through the induction of M2 macrophage polarization. BMC Microbiol..

[cit211] Narayana Iyengar S., Dietvorst J., Ferrer-Vilanova A., Guirado G., Muñoz-Berbel X., Russom A. (2021). Toward Rapid Detection of Viable Bacteria in Whole Blood for Early Sepsis Diagnostics and Susceptibility Testing. ACS Sens..

[cit212] Rashiku M. , Pandey M., and Bhattacharya S., ‘Integrating microfluidics and sensing for capability enhancement’, in Impedance Spectroscopy and its Application in Biological Detection, CRC Press, 2023, pp. 37–58, 10.1201/9781003358091-4

[cit213] Hu C. (2020). *et al.*, Disposable Paper-on-CMOS Platform for Real-Time Simultaneous Detection of Metabolites. IEEE Trans. Biomed. Eng..

[cit214] Alekhmimi N. K. (2023). *et al.*, Paper-Based Biosensor for the Detection of Sepsis Using MMP-9 Biomarker in FIP Mice Model. Biosensors.

[cit215] Chen K. (2021). *et al.*, A rapid and sensitive europium nanoparticle-based lateral flow immunoassay combined with recombinase polymerase amplification for simultaneous detection of three food-borne pathogens. Int. J. Environ. Res. Publ. Health.

[cit216] Faridi M. A., Ramachandraiah H., Banerjee I., Ardabili S., Zelenin S., Russom A. (2017). Elasto-inertial microfluidics for bacteria separation from whole blood for sepsis diagnostics. NanoBiotechnology.

[cit217] Alekhmimi N. K. (2023). *et al.*, Biosensing Platform for the Detection of Biomarkers for ALI/ARDS in Bronchoalveolar Lavage Fluid of LPS Mice Model. Biosensors.

[cit218] Damodara S., Dwivedi D. J., Liaw P. C., Fox-Robichaud A. E., Selvaganapathy P. R. (2021). Single step separation and concentration of biomarker proteins using agarose based miniaturized isoelectric gates for point of care diagnostics. Sens. Actuators, B.

[cit219] Hassan U. (2017). *et al.*, A point-of-care microfluidic biochip for quantification of CD64 expression from whole blood for sepsis stratification. Nat. Commun..

[cit220] Wei Hou H., Gan H. Y., Bhagat A. A. S., Li L. D., Lim C. T., Han J. (2012). A microfluidics approach towards high-throughput pathogen removal from blood using margination. Biomicrofluidics.

[cit221] Tanak A. S., Sardesai A., Muthukumar S., Prasad S. (2022). Simultaneous detection of sepsis host response biomarkers in whole blood using electrochemical biosensor. Bioeng. Transl. Med..

[cit222] Guo Z. (2024). *et al.*, An ultra-sensitive electrochemical biosensor for the detection of procalcitonin in sepsis patients' serum, using a Cu-BHT-based thin film. Talanta.

[cit223] Ai F., Ferreira C. A., Chen F., Cai W. (2016). Engineering of radiolabeled iron oxide nanoparticles for dual-modality imaging. Wiley Interdiscip. Rev.: Nanomed. Nanobiotechnol..

[cit224] Karageorgou M. A., Bouziotis P., Stiliaris E., Stamopoulos D. (2023). Radiolabeled iron oxide nanoparticles as dual-modality contrast agents in SPECT/MRI and PET/MRI. Nanomaterials.

[cit225] Neuwelt A., Sidhu N., Hu C. A. A., Mlady G., Eberhardt S. C., Sillerud L. O. (2015). Iron-based superparamagnetic nanoparticle contrast agents for MRI of infection and inflammation. Am. J. Roentgenol..

[cit226] Magar H. S., Hassan R. Y. A., Mulchandani A. (2021). Electrochemical impedance spectroscopy (EIS): Principles, construction, and biosensing applications. Sensors.

[cit227] Mehrban M., Madrakian T., Afkhami A., Jalal N. R. (2023). Fabrication of impedimetric sensor based on metallic nanoparticle for the determination of mesna anticancer drug. Sci. Rep..

[cit228] Chiang C. Y. (2020). *et al.*, Fiber optic nanogold-linked immunosorbent assay for rapid detection of procalcitonin at femtomolar concentration level. Biosens. Bioelectron..

[cit229] Giorgi-Coll S., Marín M. J., Sule O., Hutchinson P. J., Carpenter K. L. H. (2020). Aptamer-modified gold nanoparticles for rapid aggregation-based detection of inflammation: an optical assay for interleukin-6. Microchim. Acta.

[cit230] Fabri-Faja N. (2019). *et al.*, Early sepsis diagnosis via protein and miRNA biomarkers using a novel point-of-care photonic biosensor. Anal. Chim. Acta.

[cit231] Santopolo G., Doménech-Sánchez A., Russell S. M., De La Rica R. (2019). Ultrafast and Ultrasensitive Naked-Eye Detection of Urease-Positive Bacteria with Plasmonic Nanosensors. ACS Sens..

[cit232] Zhou X. (2022). *et al.*, Multifunctional biosensor constructed by Ag-coating magnetic-assisted unique urchin core porous shell structure for dual SERS enhancement, enrichment, and quantitative detection of multi-components inflammatory markers. Biosens. Bioelectron..

[cit233] Kundu A. (2022). *et al.*, Ultrasensitive and label-free detection of prognostic and diagnostic biomarkers of sepsis on a AgNP-laden black phosphorous-based SERS platform. Sens. Diagn..

[cit234] Stefano J. S. (2022). *et al.*, Different approaches for fabrication of low-cost electrochemical sensors. Curr. Opin. Electrochem..

[cit235] Štukovnik Z., Fuchs-Godec R., Bren U. (2023). Nanomaterials and their recent applications in impedimetric biosensing. Biosensors.

[cit236] Russell C. (2019). *et al.*, Development of a needle shaped microelectrode for electrochemical detection of the sepsis biomarker interleukin-6 (IL-6) in real time. Biosens. Bioelectron..

[cit237] Zupančič U., Jolly P., Estrela P., Moschou D., Ingber D. E. (2021). Graphene-enabled low-noise surface chemistry for multiplexed sepsis biomarker detection in whole blood. Adv. Funct. Mater..

[cit238] Singh P. , ‘Surface Plasmon Resonance: A Boon for Viral Diagnostics’, in Reference Module in Life Sciences, Elsevier, 2017, 10.1016/b978-0-12-809633-8.12245-9

[cit239] Safarzadeh M., Suhail A., Sethi J., Sattar A., Jenkins D., Pan G. (2021). A label-free dna-immunosensor based on aminated rgo electrode for the quantification of dna methylation. Nanomaterials.

[cit240] Gao J. (2017). *et al.*, A Multiplex Electrochemical Biosensor for Bloodstream Infection Diagnosis. SLAS Technol..

[cit241] Alba-Patiño A., Vaquer A., Barón E., Russell S. M., Borges M., de la Rica R. (2022). Micro- and nanosensors for detecting blood pathogens and biomarkers at different points of sepsis care. Microchim. Acta.

[cit242] Rascher D. (2014). *et al.*, Total internal reflection (TIRF)-based quantification of procalcitonin for sepsis diagnosis - A point-of-care testing application. Biosens. Bioelectron..

[cit243] Koukouvinos G., Goustouridis D., Misiakos K., Kakabakos S., Raptis I., Petrou P. (2018). Rapid C-reactive protein determination in whole blood with a White Light Reflectance Spectroscopy label-free immunosensor for Point-of-Care applications. Sens. Actuators, B.

[cit244] Sharma A. (2021). *et al.*, Optical biosensors for diagnostics of infectious viral disease: A recent update. Diagnostics.

[cit245] Bauer A., Bruegger D., Christ F. (2005). Mikrozirkulatorisches Monitoring der Sepsis. Der Anaesthesist.

[cit246] Feng X. (2020). *et al.*, The Critical Role of Tryptophan in the Antimicrobial Activity and Cell Toxicity of the Duck Antimicrobial Peptide DCATH. Front. Microbiol..

[cit247] Hoffmann J. J. M. L. (2011). Neutrophil CD64 as a sepsis biomarker. Biochem. Med..

[cit248] Accardo A., Tesauro D., Morelli G. (2013). Peptide-based targeting strategies for simultaneous imaging and therapy with nanovectors. Pharm. J..

[cit249] Yang Y. (2022). Fluorescent organic small molecule probes for bioimaging and detection applications. Molecules.

[cit250] Bhuin S., Chakraborty P., Sivasakthi P., Samanta P. K., Chakravarty M. (2023). Double-Site Twisted D-π-D′ Conjugates with Versatile Photophysical Facets for Diverse Optical Applications and Wash-Free Bioimaging of Cancer Cells. ACS Appl. Opt. Mater..

[cit251] Zhang R. R. (2017). *et al.*, Beyond the margins: Real-time detection of cancer using targeted fluorophores. Nat. Rev. Clin. Oncol..

[cit252] Layne S. P. , Bigiot I. J., Scott A. C., and Lomdahl P. S., ‘Transient fluorescence in synchronously dividing Escherichia coli (balanced growth/flavin/light scattering/nonlinear model/Raman spectroscopy)’, 1985, available: https://www.pnas.org 10.1073/pnas.82.22.7599 PMC391380 3906649

[cit253] Katz A. (2003). *et al.*, Bacteria size determination by elastic light scattering. IEEE J. Sel. Top. Quantum Electron..

[cit254] Konokhova A. I., Gelash A. A., Yurkin M. A., Chernyshev A. V., Maltsev V. P. (2013). High-precision characterization of individual E. coli cell morphology by scanning flow cytometry. Cytometry A.

[cit255] Banada P. P. (2009). *et al.*, Label-free detection of multiple bacterial pathogens using light-scattering sensor. Biosens. Bioelectron..

[cit256] Qiu L. (2021). *et al.*, Rapid detection and identification of bacteria directly from whole blood with light scattering spectroscopy based biosensor. Sens. Actuators, B.

[cit257] Damodara S., Arora J., Liaw P. C., Fox-Robichaud A. E., Selvaganapathy P. R. (2022). Single-step measurement of cell-free DNA for sepsis prognosis using a thread-based microfluidic device. Microchim. Acta.

[cit258] Chin C. D. (2011). *et al.*, Microfluidics-based diagnostics of infectious diseases in the developing world. Nat. Med..

[cit259] Battaglia F. (2022). *et al.*, Molecularly imprinted polymers as effective capturing receptors in a pseudo-ELISA immunoassay for procalcitonin detection in veterinary species. Anal. Methods.

[cit260] Kemmler M., Sauer U., Schleicher E., Preininger C., Brandenburg A. (2014). Biochip point-of-care device for sepsis diagnostics. Sens. Actuators, B.

[cit261] Park Y. (2020). *et al.*, An Integrated Plasmo-Photoelectronic Nanostructure Biosensor Detects an Infection Biomarker Accompanying Cell Death in Neutrophils. Small.

[cit262] Stefano J. S., Orzari L. O., Silva-Neto H. A., Ataíde V. N., Mendes L. F., Coltro W. K. T., Paixão T. R. L. C., Janegitz B. C. (2022). Different approaches for fabrication of low-cost electrochemical sensors. Curr. Opin. Electrochem..

[cit263] Pandey M. , Tatiya S. and Bhattacharya S., ‘Design and Development of MEMS-Based Sensors for Wearable Diagnostic Applications’, in MEMS Applications in Biology and Healthcare, AIP Publishing, 2021, pp. 1–34, 10.1063/9780735423954_010

[cit264] Martín-Fernández M. (2025). *et al.*, Circulating Extracellular Vesicle miR-150–5p as a biomarker for optimizing clinical management of sepsis and septic shock: A discovery and validation Study. J. Infect. Public Health.

[cit265] Ondevilla N. A. P. (2024). *et al.*, A point-of-care electrochemical biosensor for the rapid and sensitive detection of biomarkers in murine models with LPS-induced sepsis. Biosens. Bioelectron..

[cit266] Min J. (2018). *et al.*, Integrated Biosensor for Rapid and Point-of-Care Sepsis Diagnosis. ACS Nano.

[cit267] Tanak A. S., Sardesai A., Muthukumar S., Prasad S. (2022). Simultaneous detection of sepsis host response biomarkers in whole blood using electrochemical biosensor. Bioeng. Transl. Med..

[cit268] Lu T. C. (2024). *et al.*, Simultaneous detection of C-reactive protein and lipopolysaccharide based on a dual-channel electrochemical biosensor for rapid Gram-typing of bacterial sepsis. Biosens. Bioelectron..

[cit269] Mansor N. N. A. (2018). An amperometric biosensor for the determination of bacterial sepsis biomarker, secretory phospholipase group IIA using a tri-enzyme system. Biosens. Bioelectron..

[cit270] Tian H. Y. (2020). *et al.*, Sepsis progression monitoring via human serum fibronectin detection based on sandwich-type electrochemical immunosensor. Anal. Chim. Acta.

[cit271] Kaur I., Sharma M., Kaur S., Kaur A. (2020). Ultra-sensitive electrochemical sensors based on self-assembled chelating dithiol on gold electrode for trace level detection of copper(II) ions. Sens. Actuators, B.

[cit272] Thongkhao P., Numnuam A., Khongkow P., Sangkhathat S., Phairatana T. (2024). Disposable Polyaniline/m-Phenylenediamine-Based Electrochemical Lactate Biosensor for Early Sepsis Diagnosis. Polymers.

[cit273] Kiatamornrak P. (2022). *et al.*, A portable blood lactate sensor with a non-immobilized enzyme for early sepsis diagnosis. Analyst.

[cit274] Chen C., Gopinath S. C. B., Anbu P. (2021). Longitudinal Zeolite-Iron Oxide Nanocomposite Deposited Capacitance Biosensor for Interleukin-3 in Sepsis Detection. Nanoscale Res. Lett..

[cit275] Li J. (2023). *et al.*, Wearable and battery-free wound dressing system for wireless and early sepsis diagnosis. Bioeng. Transl. Med..

[cit276] Molinero-Fernández Á., Moreno-Guzmán M., López M. Á., Escarpa A. (2020). Magnetic bead-based electrochemical immunoassays on-drop and on-chip for procalcitonin determination: Disposable tools for clinical sepsis diagnosis. Biosensors.

[cit277] Molinero-Fernández Á., Arruza L., López M. Á., Escarpa A. (2020). On-the-fly rapid immunoassay for neonatal sepsis diagnosis: C-reactive protein accurate determination using magnetic graphene-based micromotors. Biosens. Bioelectron..

[cit278] Guillem P., Bustos R. H., Garzon V., Munoz A., Juez G. (2021). A low-cost electrochemical biosensor platform for C-reactive protein detection. Sens. Biosens. Res..

[cit279] Ge X. Y., Zhang J. X., Feng Y. G., Wang A. J., Mei L. P., Feng J. J. (2022). Label-free electrochemical biosensor for determination of procalcitonin based on graphene-wrapped Co nanoparticles encapsulated in carbon nanobrushes coupled with AuPtCu nanodendrites. Microchim. Acta.

[cit280] Wang X. Y. (2021). *et al.*, Facile construction of ratiometric electrochemical immunosensor using hierarchical PtCoIr nanowires and porous SiO2@Ag nanoparticles for accurate detection of septicemia biomarker. Bioelectrochemistry.

[cit281] Yang L., Li J. (2023). Recent advances in electrochemiluminescence emitters for biosensing and imaging of protein biomarkers. Chemosensors.

[cit282] Tanak A. S., Jagannath B., Tamrakar Y., Muthukumar S., Prasad S. (2019). Non-faradaic electrochemical impedimetric profiling of procalcitonin and C-reactive protein as a dual marker biosensor for early sepsis detection. Anal. Chim. Acta: X.

[cit283] Russell C. (2019). *et al.*, Development of a needle shaped microelectrode for electrochemical detection of the sepsis biomarker interleukin-6 (IL-6) in real time. Biosens. Bioelectron..

[cit284] Min J. (2018). *et al.*, Integrated Biosensor for Rapid and Point-of-Care Sepsis Diagnosis. ACS Nano.

[cit285] Tanak A. S., Muthukumar S., Krishnan S., Schully K. L., Clark D. V., Prasad S. (2021). Multiplexed cytokine detection using an electrochemical point-of-care sensing device towards rapid sepsis endotyping. Biosens. Bioelectron..

[cit286] Tanak A. S. (2022). *et al.*, Multiplexed host immune response biosensor for rapid sepsis stratification and endotyping at point-of-care. Biosens. Bioelectron.: X.

[cit287] Gao J. (2017). *et al.*, A Multiplex Electrochemical Biosensor for Bloodstream Infection Diagnosis. SLAS Technol..

[cit288] Sharma R., Lakshmi G. B. V. S., Kumar A., Solanki P. (2022). Polypyrrole Based Molecularly Imprinted Polymer Platform for Klebsiella pneumonia Detection. ECS Sens. Plus.

[cit289] Bonini A. (2021). *et al.*, A label-free impedance biosensing assay based on CRISPR/Cas12a collateral activity for bacterial DNA detection. J. Pharm. Biomed. Anal..

[cit290] Zhou Y., Zhang Y., Johnson A., Venable A., Griswold J., Pappas D. (2019). Detection of culture-negative sepsis in clinical blood samples using a microfluidic assay for combined CD64 and CD69 cell capture. Anal. Chim. Acta.

[cit291] Crapnell R. D., Banks C. E. (2024). Electroanalytical overview: Screen-printed electrochemical sensing platforms. ChemElectroChem.

[cit292] Lim J. M., Ryu M. Y., Kim J. H., Cho C. H., Park T. J., Park J. P. (2017). An electrochemical biosensor for detection of the sepsis-related biomarker procalcitonin. RSC Adv..

[cit293] Zelenin S., Hansson J., Ardabili S., Ramachandraiah H., Brismar H., Russom A. (2015). Microfluidic-based isolation of bacteria from whole blood for sepsis diagnostics. Biotechnol. Lett..

[cit294] Kundu S., Tabassum S., Kumar R. (2021). Plasmonic Point-of-Care Device for Sepsis Biomarker Detection. IEEE Sens. J..

[cit295] Liao J., Ren J., Wei H., Lam R. H. W., Chua S. L., Khoo B. L. (2021). Label-free biosensor of phagocytosis for diagnosing bacterial infections. Biosens. Bioelectron..

[cit296] Ganguli A. (2022). A culture-free biphasic approach for sensitive and rapid detection of pathogens in dried whole-blood matrix. Proc. Natl. Acad. Sci. U. S. A..

[cit297] Hassan U., Zhu R., Bashir R. (2018). Multivariate computational analysis of biosensor's data for improved CD64 quantification for sepsis diagnosis. Lab Chip.

[cit298] Ghonge T. (2019). *et al.*, Smartphone-imaged microfluidic biochip for measuring CD64 expression from whole blood. Analyst.

[cit299] Fang Y. L. (2021). *et al.*, An integrated microfluidic system for early detection of sepsis-inducing bacteria. Lab Chip.

[cit300] Zhou Y., Zhang Y., Johnson A., Venable A., Griswold J., Pappas D. (2019). Detection of culture-negative sepsis in clinical blood samples using a microfluidic assay for combined CD64 and CD69 cell capture. Anal. Chim. Acta.

[cit301] Yu H., Tan Y., Cunningham B. T. (2014). Smartphone fluorescence spectroscopy. Anal. Chem..

[cit302] Gordón Pidal J. M., Arruza L., Moreno-Guzmán M., López M. Á., Escarpa A. (2024). Micromotor-based dual aptassay for early cost-effective diagnosis of neonatal sepsis. Microchim. Acta.

[cit303] Gordón Pidal J. M., Arruza L., Moreno-Guzmán M., López M. Á., Escarpa A. (2023). OFF-ON on-the-fly aptassay for rapid and accurate determination of procalcitonin in very low birth weight infants with sepsis suspicion. Sens. Actuators, B.

[cit304] Mou L., Li Z., Qi J., Jiang X. (2021). Point-of-care immunoassays with tunable detection range for detecting infection in intensive care unit. CCS Chem..

[cit305] Ruppert C., Kaiser L., Jacob L. J., Laufer S., Kohl M., Deigner H. P. (2020). Duplex Shiny app quantification of the sepsis biomarkers C-reactive protein and interleukin-6 in a fast quantum dot labeled lateral flow assay. NanoBiotechnology.

[cit306] Borse V., Srivastava R. (2019). Fluorescence lateral flow immunoassay based point-of-care nanodiagnostics for orthopedic implant-associated infection. Sens. Actuators, B.

[cit307] Molinero-Fernández Á., López M. Á., Escarpa A. (2020). An on-chip microfluidic-based electrochemical magneto-immunoassay for the determination of procalcitonin in plasma obtained from sepsis diagnosed preterm neonates. Analyst.

[cit308] Ohlsson P. (2016). *et al.*, Integrated Acoustic Separation, Enrichment, and Microchip Polymerase Chain Reaction Detection of Bacteria from Blood for Rapid Sepsis Diagnostics. Anal. Chem..

[cit309] Faridi M. A., Ramachandraiah H., Banerjee I., Ardabili S., Zelenin S., Russom A. (2017). Elasto-inertial microfluidics for bacteria separation from whole blood for sepsis diagnostics. NanoBiotechnology.

[cit310] Ohlsson P. (2016). *et al.*, Integrated Acoustic Separation, Enrichment, and Microchip Polymerase Chain Reaction Detection of Bacteria from Blood for Rapid Sepsis Diagnostics. Anal. Chem..

[cit311] Choudhury B., Shinar R., Shinar J. (2004). Glucose biosensors based on organic light-emitting devices structurally integrated with a luminescent sensing element. Appl. Phys. Lett..

[cit312] Lian C., Young D., Randall R. E., Samuel I. D. W. (2022). Organic Light-Emitting Diode Based Fluorescence-Linked Immunosorbent Assay for SARS-CoV-2 Antibody Detection. Biosensors.

[cit313] Hassan U. (2017). *et al.*, A point-of-care microfluidic biochip for quantification of CD64 expression from whole blood for sepsis stratification. Nat. Commun..

[cit314] Taneja I. (2021). *et al.*, Diagnostic and prognostic capabilities of a biomarker and EMR-based machine learning algorithm for sepsis. Clin. Transl. Sci..

[cit315] Honoré A., Forsberg D., Adolphson K., Chatterjee S., Jost K., Herlenius E. (2023). Vital sign-based detection of sepsis in neonates using machine learning. Acta Paediatr..

[cit316] Gupta S., Singh A., Sharma A., Tripathy R. K. (2022). Higher Order Derivative-Based Integrated Model for Cuff-Less Blood Pressure Estimation and Stratification Using PPG Signals. IEEE Sens. J..

[cit317] Giordano M., Dheman K., Magno M. (2025). SepAl: Sepsis Alerts on Low-Power Wearables With Digital Biomarkers and On-Device Tiny Machine Learning. IEEE Sens. J..

[cit318] Graziani A. C. (2017). *et al.*, High efficiency binding aptamers for a wide range of bacterial sepsis agents. J. Microbiol. Biotechnol..

[cit319] Zeng H. (2013). *et al.*, Optical signature of symmetry variations and spin-valley coupling in atomically thin tungsten dichalcogenides. Sci. Rep..

[cit320] Xu L. (2020). *et al.*, Accurate MRSA identification through dual-functional aptamer and CRISPR-Cas12a assisted rolling circle amplification. J. Microbiol. Methods.

[cit321] Fukuzumi N., Nakagawa T., Hirao G., Ogawa A., Maeda M., Asahi T., Zako T. (2025). Detection of C-reactive Protein Using Single Cluster Analysis of Gold Nanoparticle Aggregates Using a Dark-Field Microscope Equipped with a Smartphone. Sens. Diagn..

[cit322] Singer M. (2016). *et al.*, The third international consensus definitions for sepsis and septic shock (Sepsis-3). JAMA.

[cit323] Ferreira I. M., Lacerda C. M. d. S., Santos S. R. d., Barros A. L. B. d., Fernandes S. O., Cardoso V. N., Andrade A. S. R. d. (2017). Detection of bacterial infection by a technetium-99m-labeled peptidoglycan aptamer. Biomed. Pharmacother..

[cit324] Rudd K. E. (2018). *et al.*, The global burden of sepsis: Barriers and potential solutions. Crit. Care.

[cit325] Sundriyal P., Pandey M., Bhattacharya S. (2020). Plasma-assisted surface alteration of industrial polymers for improved adhesive bonding. Int. J. Adhes. Adhes..

[cit326] Pandey M., Mairal A., Gupta H., Kumar A., Bhattacharya S. (2024). Rapid surface modification of PEEK by ambient temperature sulfonation for high shelf-life biomedical applications. Surf. Interfaces.

[cit327] Kumar S., Bhushan P., Pandey M., Bhattacharya S. (2019). Additive manufacturing as an emerging technology for fabrication of microelectromechanical systems (MEMS). J. Micromanufacturing.

